# Onion-like networks are both robust and resilient

**DOI:** 10.1038/s41598-018-29626-w

**Published:** 2018-07-26

**Authors:** Yukio Hayashi, Naoya Uchiyama

**Affiliations:** Japan Advanced Institute of Science and Technology, Graduate School of Advanced Institute of Science and Technology/Division of Transdisiplinary Sciences, Ishikawa, 923-1292 Japan

## Abstract

Tolerant connectivity and flow transmission within capacity are crucial functions as network. However, the threats to malicious attacks based on intelligent node selections and rapid breakdown by cascading overload failures increase more and more with large blackout or congestion in our contemporary networking systems and societies. It has been recently suggested that interwoven loops protect the network functions from such damages, but it is a computationally intractable combinatorial problem to maximize a set of necessary nodes for loops in order to improve the robustness. We propose a new method by enhancing loops in the incremental growth for constructing onion-like networks with positive degree-degree correlations, whose topological structure has the optimal tolerance of connectivity against attacks in the state-of-the-art. Moreover, we find out that onion-like networks acquire adaptive capacity in resilience by a change of routing policy for flow control to absorb cascading overload failures triggered by a single attack and simultaneous multi-attacks. The inhibitory effect is stronger than that in scale-free networks found in many real systems.

## Introduction

It is well-known that there exist a common topological structure called scale-free (SF) in many real social, technological, biological networks, and they are extremely vulnerable against intentional attacks to large degree nodes of hubs^1^. When similar degree nodes tend to connect in a SF network, the connectivity gives the optimal attack tolerance^2,3^ under its power-law degree distribution. Such networks with positive degree-degree correlations are called onion-like because of visualizing as similar degree nodes locate on concentric circles in decreasing order of degrees from core to peripheral. Onion-like networks can be constructed by self-organized incrementally growing methods^4–6^ instead of whole rewiring^7^ for positive degree-degree correlations. Other intelligent attacks to influencer^8^ or feedback vertex set (FVS)^9^ recently appear, and the insistent destruction of loops give severer damages than the conventionally worst hub attacks. However, one of the self-organized growing method^6^ takes into account the weakness inversely by enhancing loops in simple link attachments on the growth. Influencer and FVS are the minimum set of nodes to maximally prevent information spreading and formation of loops (or referred to cycles), respectively, as these nodes are removed in a network.

On the other hand, from the asymptotic equivalence of dismantling and decycling problems at infinite graphs in a large class of random networks with light-tailed degree distribution^10^, the strong robustness may be related to increasing the size of FVS which is necessary to form loops. In other words, the existence of many loops is probably crucial to maintain the connectivity of network within a finite size. Here, dismantling (or decycling) problem is to find the minimum set of nodes if its removal yields a graph with the largest connected cluster whose size is at most a constant (or a graph without loops). However, it is a nondeterministic polynomial(NP)-hard problem to find FVS^11^, there is no efficient algorithm for the exact solution due to the worst case difficulty. Thus, we consider a heuristic method for increasing the size of FVS, and show a further improvement of robustness in growing onion-like networks than the previous method^6^. We emphasize that onion-like networks emerge through enhancing loops with indirect influence to degree-degree correlations.

Moreover, we reveal the resilient property for onion-like networks. As the major meanings of resilience in system science, it is pointed out that^12^ *resilience is the ability to prevent something bad from happening, or the ability to prevent something bad from becoming worse, or the ability to recover from something bad once it has happened*, which focus on the buffer capacity to absorb shocks and still maintain the functions. We wish to head for the adaptive capacity, because *resilience of complex adaptive system is not simply about resistance to change and conservation of existing systems, but also about opportunities that disturbance opens up in terms of recombination of evolved structures and process, renewal of the system and emergence of new trajectories*^13^. For example, after a happened damage and the succeeding failures, a change of routing polity form usual shortest-based to congestion-aware load-based selection of paths does not mean complete recovery but corresponds to renewal in flow control process. Thus, we show that onion-like network structure supplely absorb and decentralize pressure of transfer flow in the above change of routing policy, whose flow control inhibits cascading overload failures much more than the conventional defense^14^ and navigation^15^ strategies. In this paper, we mainly discuss the improvement in resilience achieved by adaptive changes in transmission dynamics rather than quick changes in the network structure, however the high performance is supported by the topological existence of many bypasses originated from interwoven loops. Indeed, even for a same routing strategy, some differences appear in comparison with SF and onion-like networks as shown later. These results will open a prospective direction to develop more resilient structure in future re-organizing networks than SF structure found in many real systems.

## Results

### Incrementally growing onion-like networks

**Figure 1:**
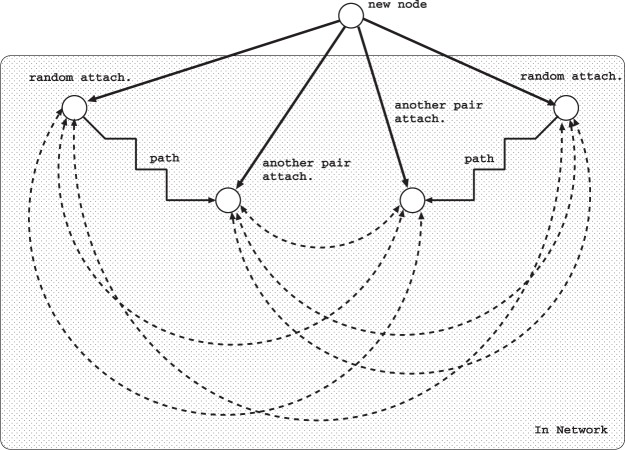
Pairs of attachments in RLD or MED. Bold and dashed lines denote added links to a current network and existing paths in the network. Zigzag lines denote paths of *μ* = 5 intermediations from randomly attached nodes.

We consider incrementally growing methods of strongly robust onion-like networks with positive degree-degree correlations^2,3^ by the following attachments via intermediations and new modifications of the minimum degree selection. At each time step of growing from an initial configuration until reaching a size *N*: total number of nodes, a new node is added and connects to existing nodes. As the connection rule for even number *m* links emanated from the new node, we introduce a pair of attachments based on random and long-distance (RLD) attachment, from which a range-limited approximation of RLD referred to as intermediation (MED) attachment^6^ is derived. We assume that each link is undirected. Since multiple links are prohibited in the attachments from a new node, if a same node is chosen, then other selection is tried again. We should remark that loops and bypasses originated from them are formed by pairs of attachments as shown in Fig. 1. The interwoven loops via new node are significant for *m* ≥ 4^6^.

RLD-kmin: One of link destination is uniformly randomly chosen as encountering, and another link destination is the furthest node from the randomly chosen pair node. When there are several candidates of the furthest with a same distance counted by hops, the node with the minimum degree is selected.

MED-kmin: Instead of the furthest node, we consider intermediations in a few hops as a range-limited approximation to reduce connection cost or effort. We select a node with the minimum degree for intermediations in *μ* hops from the randomly chosen pair node. Intermediations in *μ* hops mean attachments to the *μ* + 1-th neighbors.

MED-rand: Instead of the node with the minimum degree, we randomly chosen a node in the *μ* + 1-th neighbors from the randomly chosen pair node. This is the previous best method in growing strongly robust onion-like networks^6^.

Since older nodes tend to have larger degrees in random attachment^16^, this attachment contributes to making positive correlations among large degree nodes, while another attachment to the node with the minimum degree enhances positive correlations among low degree nodes. In other words, the attachment establishes a connection between the node with the minimum degree in the *μ* + 1-th neighbors and a new node with the minimum degree *m* in the network, then it enhances positive correlations among low degree nodes. When a node is randomly chosen in the *μ* + 1-th neighbors in the previous method^6^, the degree of randomly chosen node is usually larger than the minimum in the neighbors. Therefore, the correlations become somewhat weaker in MED-rand than MED-kmin from the difference of attached nodes with the minimum and larger degrees by a new node. This modification from random selection to minimum degree selection in the neighbors seems to be slight, however it is very important to improve the robustness of connectivity as shown later.

We also consider other attachments for increasing the size of FVS in order to study the effect of loops on the robustness, since the nodes of FVS are necessary to form loops. We discuss not exact nodes of FVS but the candidates by an approximation method^9,17^ because of its NP-hardness^11^. To investigate the potential of FVS for the robustness of connectivity especially in onion-like networks, we consider the following four types of attachments for direct links from a new node or pairs of nodes with/without recalculation of ${q}_{i}^{0}$ for Eqs (2–6) in the approximation method. Note that the probability that a node is included in FVS is lower as it has smaller ${q}_{i}^{0}$. By these attachments, the selected nodes with small ${q}_{i}^{0}$ as the link destinations newly contribute to forming loops via a new node. Thus, the attached nodes may be joined in FVS, the enhancement of robustness is expected.

All-minq-recal: As a link destination, the node with the minimum ${q}_{i}^{0}$ is chosen and directly attached from a new node. This process is repeated in *m* times through the recalculation of ${q}_{i}^{0}$ after every selection of the attached node.

All-mimq-bottom4: The nodes of the bottom *m* in increasing order from the minimum ${q}_{i}^{0}$ are chosen and directly attached from a new node. The value of ${q}_{i}^{0}$ is not recalculated.

Rminq-recal: Pairs of nodes are attached from a new node in *m*/2 times. One of link destination is uniformly randomly chosen, and another link destination is the chosen node with the minimum ${q}_{i}^{0}$ in the *μ* + 1-th neighbors of pair node through the recalculations of ${q}_{i}^{0}$ after every selection of pair.

Rminq-norecal: As the half of destinations, *m*/2 nodes are randomly chosen in advance. Then, another link destination is the chosen node with the minimum ${q}_{i}^{0}$ in the *μ* + 1-th neighbors of each pair of random selection. The set $\{{q}_{i}^{0}\}$ is calculated only at once.

Once $\{{q}_{i}^{0}\}$ is calculated after the updating in appropriate number of rounds ≈100 by the massage-passing of Eqs (2–6), the minimum ${q}_{i}^{0}$ and the bottom *m* nodes are easily obtained. Even if the attached nodes are chosen in the *μ* + 1-th neighbors, the calculations are necessary for the whole *N* nodes. While many recalculations of $\{{q}_{i}^{0}\}$ to grow a network are computationally expensive, we study the attachments with recalculations to compare the robustness with that in the networks generated by other attachments.

### Further improved robustness in growing onion-like networks

We show an improvement of robustness from the previous results^6^ for growing onion-like networks with positive degree-degree correlations. To investigate degree-degree correlations, we measure the assortativity −1 ≤ *r* ≤ 1 as the Pearson correlation coefficient for degrees^18^.

$$r\mathop{=}\limits^{{\rm{def}}}\frac{4M\sum _{e}({k}_{e}{k^{\prime} }_{e})-{[\sum _{e}({k}_{e}+{k^{\prime} }_{e})]}^{2}}{2M\sum _{e}({k}_{e}^{2}+{k^{\prime} }_{e}^{2})-{[\sum _{e}({k}_{e}+{k^{\prime} }_{e})]}^{2}},$$

where *k*_*e*_ and *k*′_*e*_ denote degrees at end-nodes of link *e*, *M* is the total number of links. The positive or negative correlation is distinguished by the sign *r* > 0 or *r* < 0.

In addition, to investigate robustness of connectivity we use the most commonly used measure: robustness index^3^

$$R\mathop{=}\limits^{{\rm{def}}}\sum _{q=\mathrm{1/}N}^{1}S(q)/N,$$

where *S*(*q*) denotes the number of nodes included in the giant component (GC as the largest connected cluster) after removing *qN* nodes, *q* is a fraction of removed nodes by intelligent High Degree Adaptive (HDA) attacks^8^ with recalculation of the highest degree node as the target, or Belief Propagation (BP) attacks^9^ with recalculation of the highest ${q}_{i}^{0}$ by Eqs (2–6) in a network. To simplify the discussion, we omit Collective Influence attacks^8^ because it gives intermediate damage between the typical HDA and the worst BP attacks^6^. If a network has both high *R* and *r* values by investigating these measures, it belongs to an onion-like network. Because connections among similar degree nodes in an onion-like network give rise to a high *r* value with positive degree-degree correlations, and consequently emerge the strong robustness with a high *R* value^2,3^, which is not so much affected by the rewiring^7^ for enhancing degree-degree correlations (as mentioned later in Table 1). There exists a robust network with only high *R* but low $r\lesssim 0$, which is not onion-like due to non-positive correlations^6^. In the following, we set *m* = 4 links for attachments from a new node in order to effectively enhance the robustness by interwoven loops.

**Table 1:** Robustness index against BP and HDA attacks in the networks at *N* = 5000 grown with *m* = 4 links per time from the initial complete graph *K*_5_.

Network	*R*_*bp*_Ori	*R*_*bp*_Rew	*R*_*hub*_Ori	*R*_*hub*_Rew
MED-kmin-*μ*0	0.361550	0.359178	0.370585	0.376964
MED-kmin-*μ*1	0.352980	0.364312	0.366646	0.382378
MED-kmin-*μ*2	0.360434	0.366723	0.374655	0.384626
MED-kmin-*μ*3	0.359403	0.367581	0.373487	0.385679
MED-kmin-*μ*4	0.359551	0.367752	0.373386	0.385714
BA model	0.223078	0.316637	0.229811	0.335264

Ori and Rew denote the original networks by MED-kmin or BA model and the rewired versions, respectively. The case of Ori for *μ* = 1 has slightly smaller *R* than other cases of Ori for *μ* = 0, 2, 3, 4 in MED-kmin. These results are averaged over 100 realizations.

**Figure 2:**
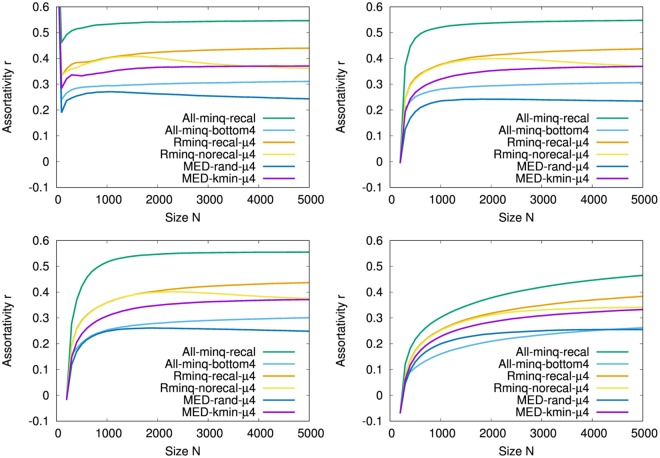
Assortativity *r* for size *N* in onion-like networks grown with *m* = 4 links per time step from typical initial configurations. (Top Left) Initial configurations of complete graph *K*_5_ among five nodes, (Top Right) ER random graph with Poisson degree distribution, (Bottom Left) random attachment network with exponential degree distribution, and (Bottom Right) SF network by BA model of 200 nodes. Note that *K*_5_ has *r* = 1.0 at *N* = 5. These results are averaged over 100 realizations.

Figure 2 shows the assortativity *r* in the growing networks by the attachments of All-minq-recal, All-minq-bottom4, Rminq-recal-*μ*4, Rminq-norecal-*μ*4, MED-rand-*μ*4, and MED-kmin-*μ*4 in *μ* = 4 intermediations from four types of the typical initial configurations: complete graph among five nodes, Erdös–Rényi (ER) random graph with Poisson degree distribution, random attachment network with exponential degree distribution, and SF network by Barabási-Albert (BA) model^16^. To be uncorrelated in the initial networks, we add the procedure of configuration model by uniformly random rewiring^19^ under these degree distributions. As shown in Fig. 2, All-minq-recal (green line) has strong degree-degree correlations, while All-minq-bottom4 (light blue line) and MED-rand-*μ*4 (blue line) have slightly weak but positive correlations. MED-kmin-*μ*4 (purple line) has moderate *r* > 0.3, which corresponds to onion-like networks. Remember that too large positive correlations are not suitable to be robust^2^. In comparison with same color lines, the dependency of the initial configurations is very small except the initial SF networks whose case takes a larger size for the convergence in the growth.

**Figure 3:**
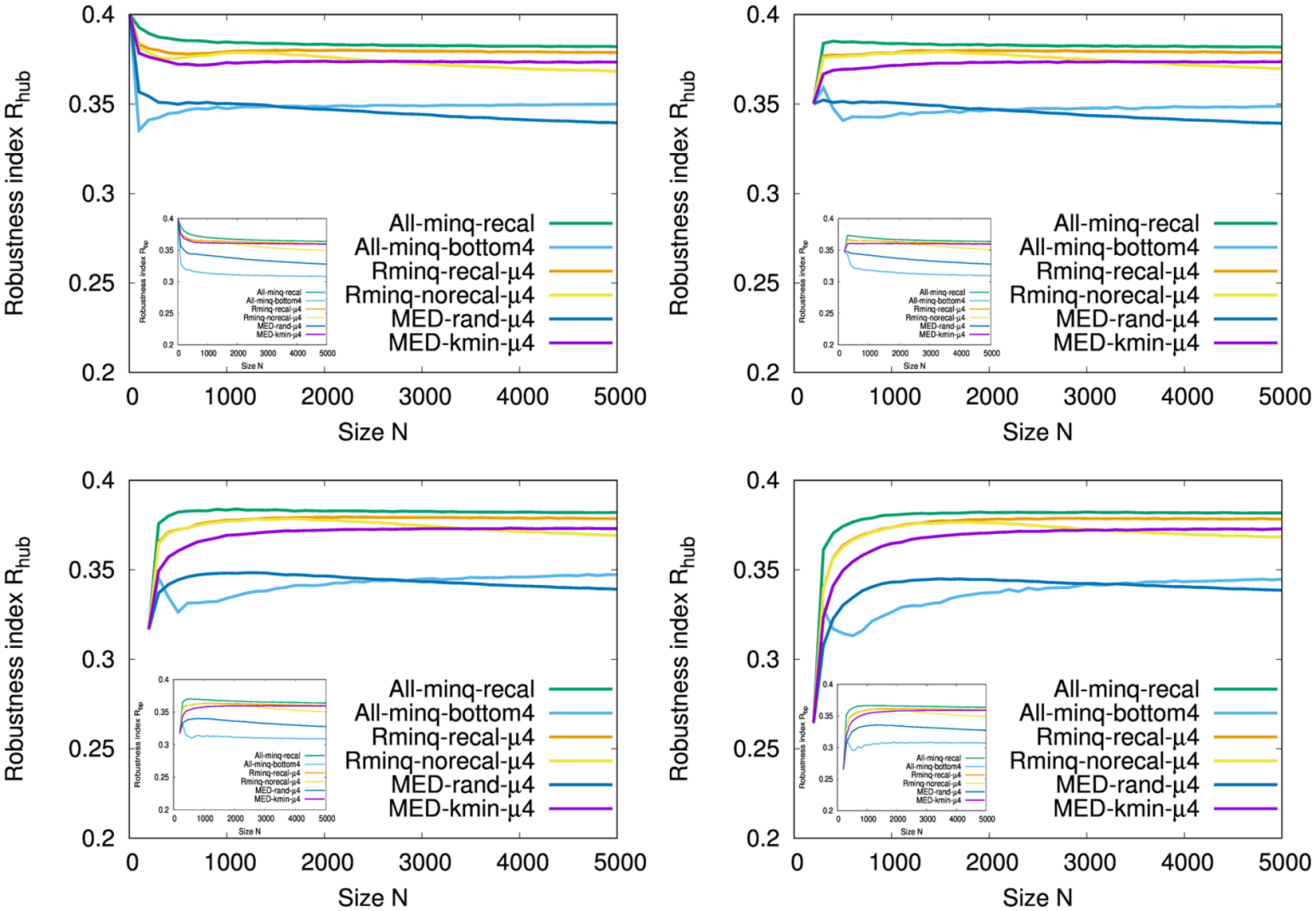
Robustness index *R* against HDA attacks (Inset BP attacks) in onion-like networks for size *N* grown with *m* = 4 links per time step from typical initial configurations. (Top Left) Initial configurations of complete graph *K*_5_ among five nodes, (Top Right) ER random graph with Poisson degree distribution, (Bottom Left) random attachment network with exponential degree distribution, and (Bottom Right) SF network by BA model of 200 nodes. These results are averaged over 100 realizations.

Figure 3 and the Inset show the robustness index *R* against HDA and BP attacks, respectively, in the growing networks by the attachments from the typical initial configurations. Note that initial complete graph has the maximum value of *R* = 0.5 at *N* = 5. It suggests the crucial importance for increasing the size of FVS that All-minq-recal (green line) and Rminq-recal-*μ*4 (orange line) have the largest *R* for both HDA and BP attacks, however they require much computation for selecting the attached nodes in constructing the networks. While MED-kim-*μ*4 (purple line) has a similar large *R* to them, All-minq-bottom4 (light blue line) and MED-rand-*μ*4 (blue line) as the previously best method^6^ have slightly smaller *R*. Therefore, newly proposed MED-kim-*μ*4 (purple line) is the most suitable with high robustness but less computation.

**Figure 4:**
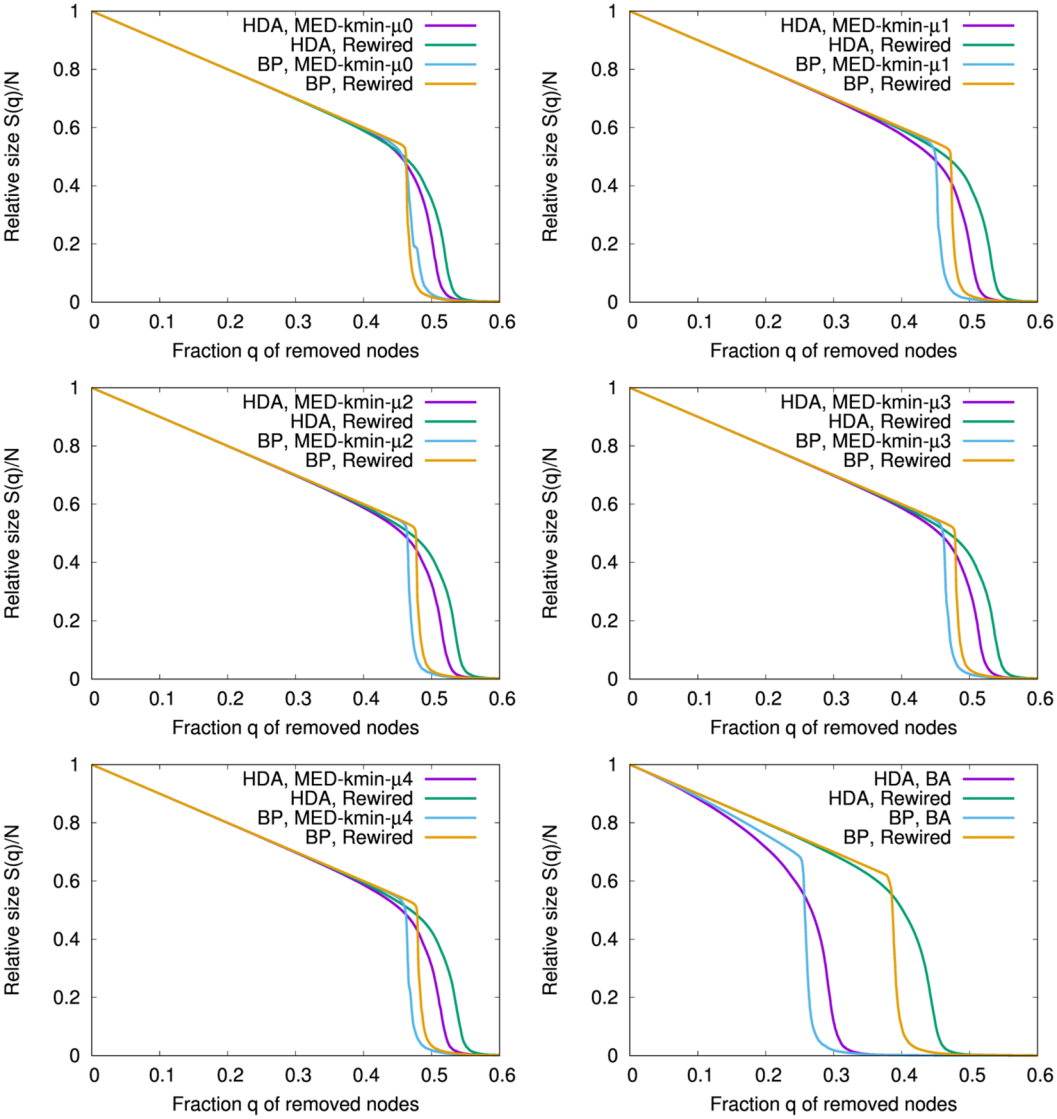
Robustness against HDA and BP attacks. (Left from Top to Bottom) Onion-like networks by MED *μ* = 0, 2, 4, (Right from Top to Bottom) Onion-like networks by MED *μ* = 1, 3 and SF networks by BA model. These results are averaged over 100 realizations.

It is common for all cases that the behavior of *R* become stable with high values in the early stage of *N* < 1000 in Fig. 3 and the Inset. Moreover, as mentioned in^6,9^, BP attacks give larger damage than HDA attacks because of *R*_*bp*_ < *R*_*hub*_ in comparison with same color lines corresponded in Fig. 3 and the Inset. Figure 4 shows the relative size *S*(*q*)/*N* for a fraction *q* of removed nodes by HDA and BP attacks on the networks at *N* = 5000 grown from the initial complete graph *K*_5_ in comparison with that in the rewired version^7^ as the nearly optimal attack-tolerance under a given degree distribution. In our onion-like networks by MED-kmin the differences for the rewired versions are very small, while in SF networks by BA model there are large gaps between the original (green, orange line) and the rewired version (purple, light blue line). The small gap (between purple and green lines, light blue and orange lines) means that the rewiring is no longer effective to enhance degree-degree correlations in our network, because similar degree nodes are already connected in it. In contrast, the large gap means that the rewiring is effective to improve the robustness by enhancing degree-degree correlations in SF network under its power-law degree distribution. Note that the rewiring generates a onion-like topological structure from any original network which may be not onion-like. The results of robustness index are summarized in Table 1 to compare the original with the rewired version.

**Figure 5:**
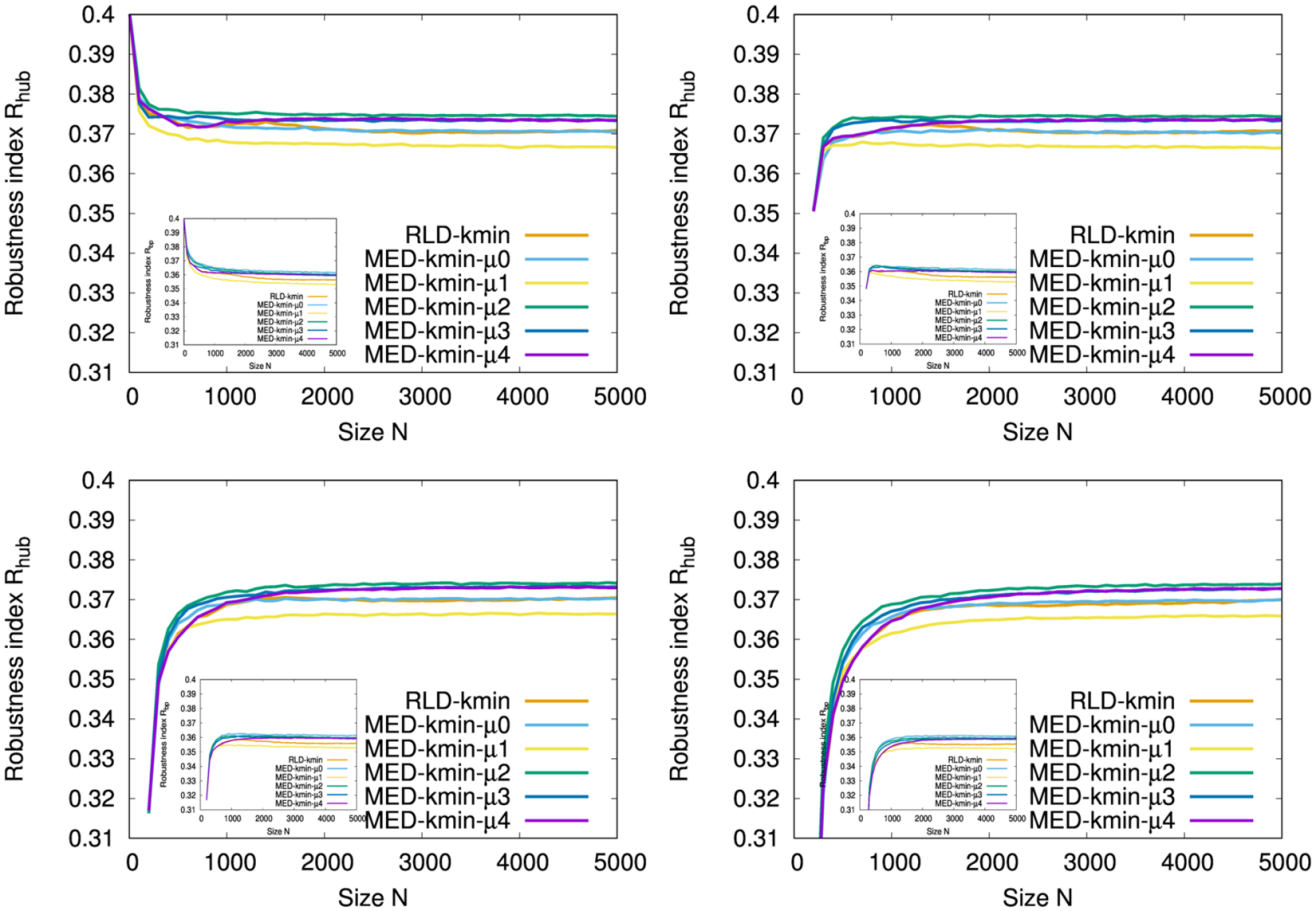
Comparison of robustness index *R* against HDA attacks (Inset BP attacks) in MED-kmin and RLD-kmin in the evolution of onion-like networks grown with *m* = 4 links per time step from the initial configurations. (Top Left) Initial configurations of complete graph *K*_5_ among five nodes, (Top Right) ER random graph with Poisson degree distribution, (Bottom Left) random attachment network with exponential degree distribution, and (Bottom Right) SF network by BA model of 200 nodes. These results are averaged over 100 realizations.

**Figure 6:**
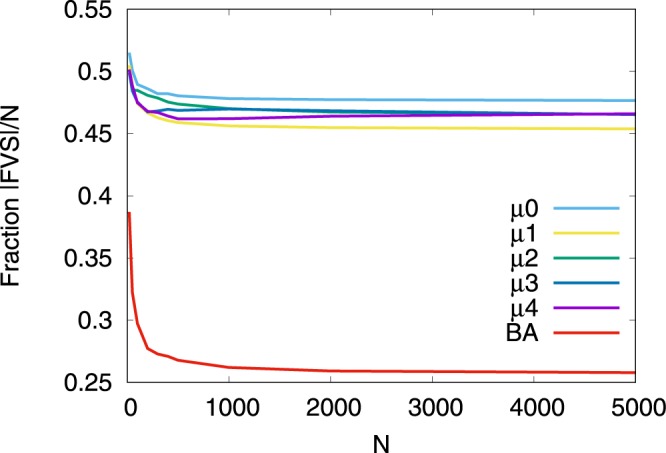
Fraction of the size of FVS in growing onion-like networks by MED-kmin and SF networks by BA model from the initial complete graph *K*_5_. These results are averaged over 100 realizations. Each fraction also shows the critical *q* value with breakdown of GC for *N* = 5000 in Fig. 4.

In the growing networks by MED-kmin attachment, we compare the robustness for the number *μ* of intermediations as shown in Fig. 5 and the Inset. The differences between the initial configurations from Top Left to Bottom Right are little for *N* > 500. For HDA attacks in Fig. 5, MED-kmin-*μ*2 (green line) has the largest *R*. The order of higher *R* is MED-kmin-*μ*2 (green line) > MED-kmin-*μ*4 (purple line) ≈ MED-kmin-*μ*3 (blue line) > MED-kmin-*μ*0 (light blue line) > RLD-kmin (orange line) > MED-kmin-*μ*1 (yellow line). For BP attacks in the Inset of Fig. 5, MED-kmin-*μ*0 (light blue line) has the largest *R*. The order of higher *R* is MED-kmin-*μ*0 (light blue line) > MED-kmin-*μ*2 (green line) > MED-kmin-*μ*3 (blue line) ≈ MED-kmin-*μ*4 (purple line) > RLD-kmin (orange line) > MED-kmin-*μ*1 (yellow line), which is slightly different from the order for HDA attacks. This order is corresponding to the decreasing order of fractions of FVS estimated by the approximation method^17^ for *μ*0, *μ*2, *μ*3, *μ*4, *μ*1 as shown in Fig. 6 (from top to bottom lines), and suggests a network becomes more robust as larger fractions of FVS (see Supplementary information, S1). These results show that the attachment to the furthest node by RLD-kmin is not necessary, rather the attachment to a distant node in a few hops by MED-kmin is better to be robust network. There remains a question why MED-kmin-*μ*1 is the worst in this study, however the degradation of *R* is very small.

### Resilience to absorb overload by flow control

We introduce a model of cascading overload failures^14^, which can be widely applied for communication or transportation networks with routing flows. For a given (undirected) network of a constant size *N*, we assume that at each time step a communication request is generated between every pair of nodes (*i*, *j*) and a packet (unit object for transfer) is transmitted along paths connecting nodes *i* and *j*. The selections of paths are depending on routing strategies explained later. The load *L*_*k*_(*t*) of node *k* at time *t* is defined by the total amount of packets passing through the node *k* per unit time. In the case of shortest paths counted by hops, the load is nothing but the betweenness centrality. The load capacity *C*_*i*_ of node *i* is set to be proportional to its initial and necessary load *L*_*i*_(0),

1 $${C}_{i}\mathop{=}\limits^{{\rm{def}}}\mathrm{(1}+\alpha ){L}_{i}\mathrm{(0),}$$

where a constant *α* ≥ 0 is the tolerance parameter. We assume that before cascading failures the initial paths are usual shortest paths as a base for comparison with routing strategies.

Cascading overload failures may occur from a small trigger through the following process.

Step 0: An initial attack, e.g. to the node with the maximum degree or the maximum load, is given at *t* = 0. After the initial attack, the damaged node and the links emanated from it are removed.

Step 1: At next time *t* ← *t* + 1, by changing paths due to the trigger of attack or the repeatedly succeeding node failures, the loads {*L*_*i*_(*t*)} of affected nodes are updated. If some nodes receive much loads that exceed own capacities, then the overloaded nodes collapse, and are removed as malfunction.

Step 2: Until no failures are propagated, go to Step 1. The cascading process is stopped at *T* when the updated load satisfies *L*_*k*_(*T*) ≤ *C*_*k*_ for all remaining *N*′ nodes.

The damage is quantified by the relative size $G\mathop{=}\limits^{{\rm{def}}}N^{\prime} /{{\rm N}}$ of the GC for varying the value of tolerance parameter *α*. Simultaneously, we measure the network efficiency

$$E\mathop{=}\limits^{{\rm{def}}}\frac{1}{N(N-\mathrm{1)}}\sum _{i\ne j}\frac{1}{{D}_{ij}},$$

where *D*_*ij*_ denotes the length of shortest path counted by hops between nodes *i* and *j*. Note that 1/*E* is the harmonic mean of path lengths between two nodes in a network, and slightly underestimated as smaller than the arithmetic mean.

**Figure 7:**
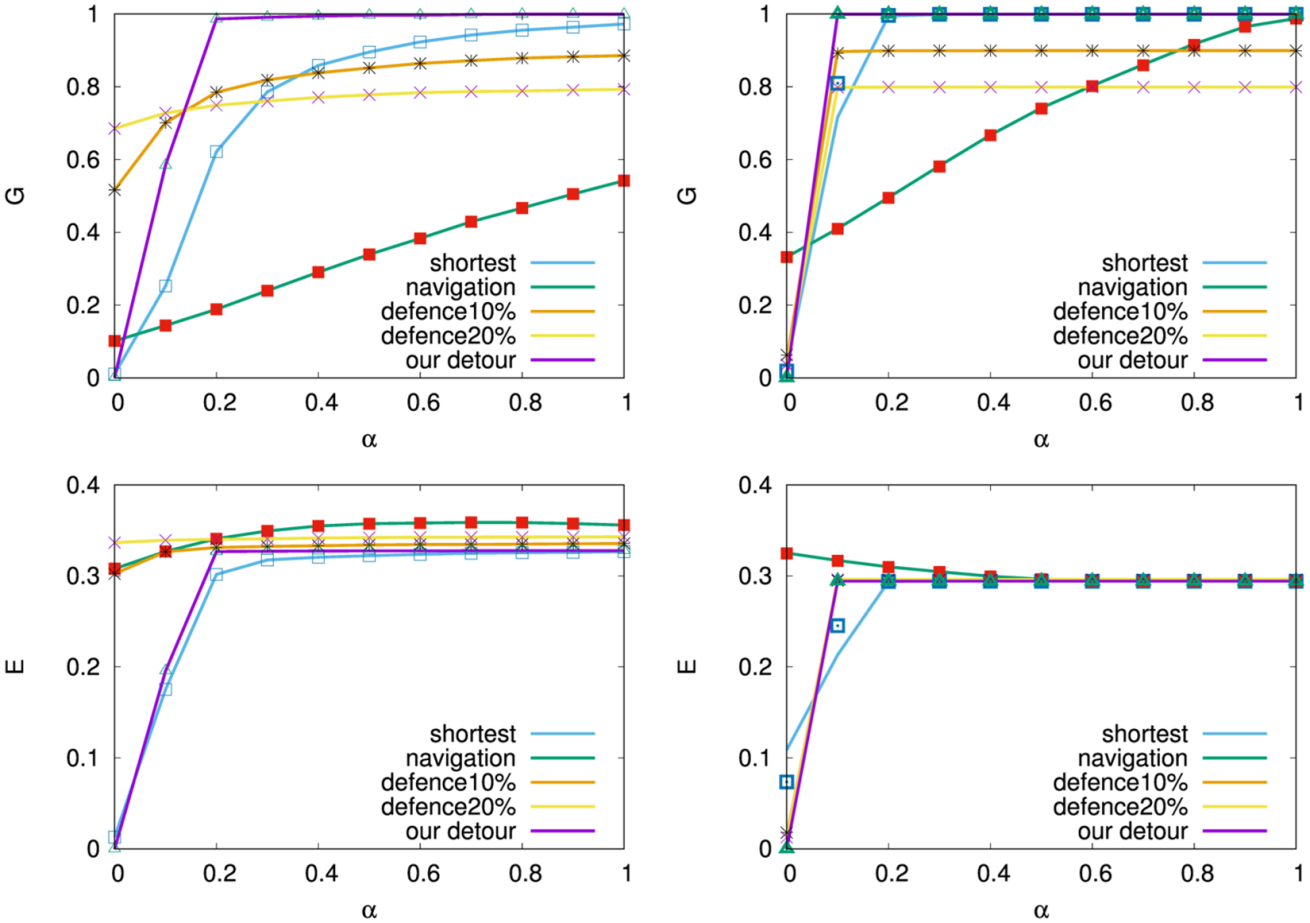
Cascading failures measured by relative size *G* and network efficiency *E* for tolerant parameter *α*. Trigger by a removal node with maximum degree or maximum load is distinguished by line or mark. (Left) SF networks by BA model, (Right) Onion-like networks by MED-kmin *μ*4. For other *μ* = 0, 1, 2, 3, similar results are obtained (See also Fig. 8).

**Figure 8:**
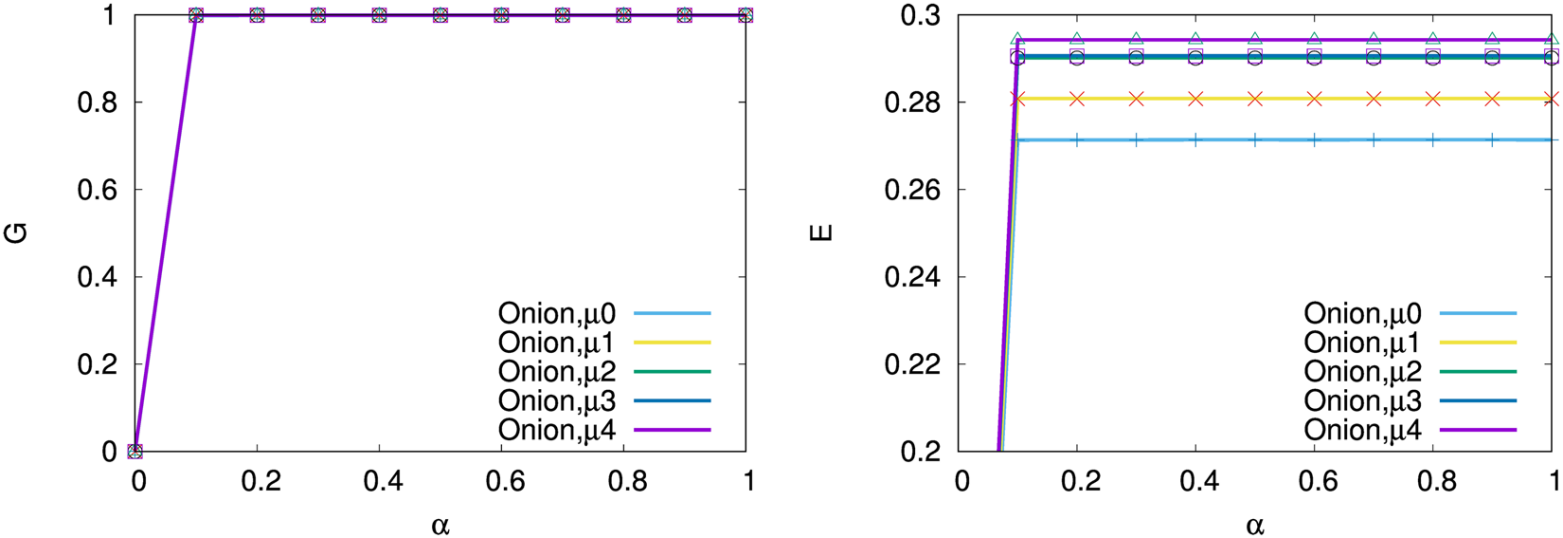
Comparison of G & E in cascading failures for our detour routing on onion-like networks by MED-kmin of *μ* = 0, 1, 2, 3, 4 intermediations.

In the following, each result is averaged over 10 realizations at *N* = 10^3^ for SF networks by BA model and onion-like networks by MED-kmin grown from a complete graph *K*_5_, since the variance is almost smaller than 10^−6^ and the amount of computation is huge for many combinations. As shown in Fig. 7, more nodes survive in a larger *G* by our detour routing (purple line) than the usual shortest-based routing (light blue line), the navigation (green line)^15^, and the defense (orange and yellow lines) strategies with *f*_*s*_ = 0.1 or 0.2 (10 or 20% sacrifices)^14^, while high network efficiency *E* is obtained for *α* > 0.2 in each of them. Note that the value of *E* is recovered to the original levels: 0.333, 0.271, 0.281, 0.290, 0.291, 0.294, in the non-damaging networks generated by BA model and MED-kmin of *μ* = 0, 1, 2, 3, 4, respectively. In particular, the inhibitory effect on cascading failures is superior in onion-like networks (right of Fig. 7) which have a larger *G* and just a little lower *E* than the corresponding results in SF networks (left of Fig. 7). We remark that the defense (orange and yellow lines) strategy is no longer effective because of a very small *G* for *α* < 0.1 in onion-like networks. There is little difference for the trigger nodes with the maximum degree and load distinguished by line and mark. The results in the right of Fig. 7 are investigated in more detail for our detour routing on onion-like networks generated by MED-kmin. As shown in Fig. 8, the relative size *G* is coincident in varying $\mu =0\sim 4$, while the network efficiency *E* is higher as the number *μ* of intermediations is larger. The reason of high network efficiency is because many shortest paths are detected in our routing as shown in Table 2.

**Table 2:** Rate of including the shortest paths in our routing on SF and onion-like networks by BA model and MED-kmin over 100 realizations.

*α*	SF	Onion *μ*0	*μ*1	*μ*2	*μ*3	*μ*4
0.0	0.94506	0.99284	0.9949	0.99257	0.99457	0.9952
0.1	0.94506	0.99284	0.9949	0.99256	0.99457	0.9952
0.2	0.94507	0.99284	0.9949	0.99257	0.99457	0.9952
0.3	0.94506	0.99284	0.9949	0.99256	0.99457	0.9952
0.4	0.94506	0.99284	0.9949	0.99256	0.99457	0.9952
0.5	0.94507	0.99284	0.9949	0.99256	0.99457	0.9952
0.6	0.94507	0.99284	0.9949	0.99256	0.99457	0.9952
0.7	0.94506	0.99284	0.9949	0.99256	0.99458	0.9952
0.8	0.94506	0.99284	0.9948	0.99256	0.99457	0.9952
0.9	0.94507	0.99284	0.9949	0.99256	0.99457	0.9952
1.0	0.94507	0.99284	0.9948	0.99256	0.99458	0.9952

When there exist some paths between two nodes in our routing, we consider the fraction of coincidences of the number of hops in our detour paths and the shortest paths. The rate is accumulated by the fractions for all combinations of source and terminal nodes.

In addition, for different distributions of capacity ${C}_{i}\mathop{=}\limits^{{\rm{def}}}{L}_{i}(0)$ + *α*′*L*_*i*_(0)^*β* ^^20^ and ${C}_{i}\mathop{=}\limits^{{\rm{def}}}(1+\alpha ^{\prime} {(\frac{{k}_{i}}{{k}_{max}})}^{\beta }){L}_{i}\mathrm{(0)}$^21,22^, we obtain the advantage of our routing to other strategies as similar to Figs 7 and 8 (see Supplementary information, S3), where *α*′ is set as $\alpha {\sum }_{i}{L}_{i}\mathrm{(0)/}{\sum }_{i}{L}_{i}{\mathrm{(0)}}^{\beta }$ and $\alpha {\sum }_{i}{L}_{i}\mathrm{(0)/}{\sum }_{i}{({k}_{i}/{k}_{max})}^{\beta }{L}_{i}\mathrm{(0)}$, respectively, for 0 ≤ *α* ≤ 1 and 0.2 ≤ *β* ≤ 1.4 in order to be equivalent to the total capacity of load ${\sum }_{i}{C}_{i}$ defined by Eq. (1). Thus, our detour routing according to Eqs (7 and 8) distributes flow in the way to avoid passing through nodes with much load, the resilient effect is analogous to decentralization of physical force in a shock absorber for a given impact pressure.

**Figure 9:**
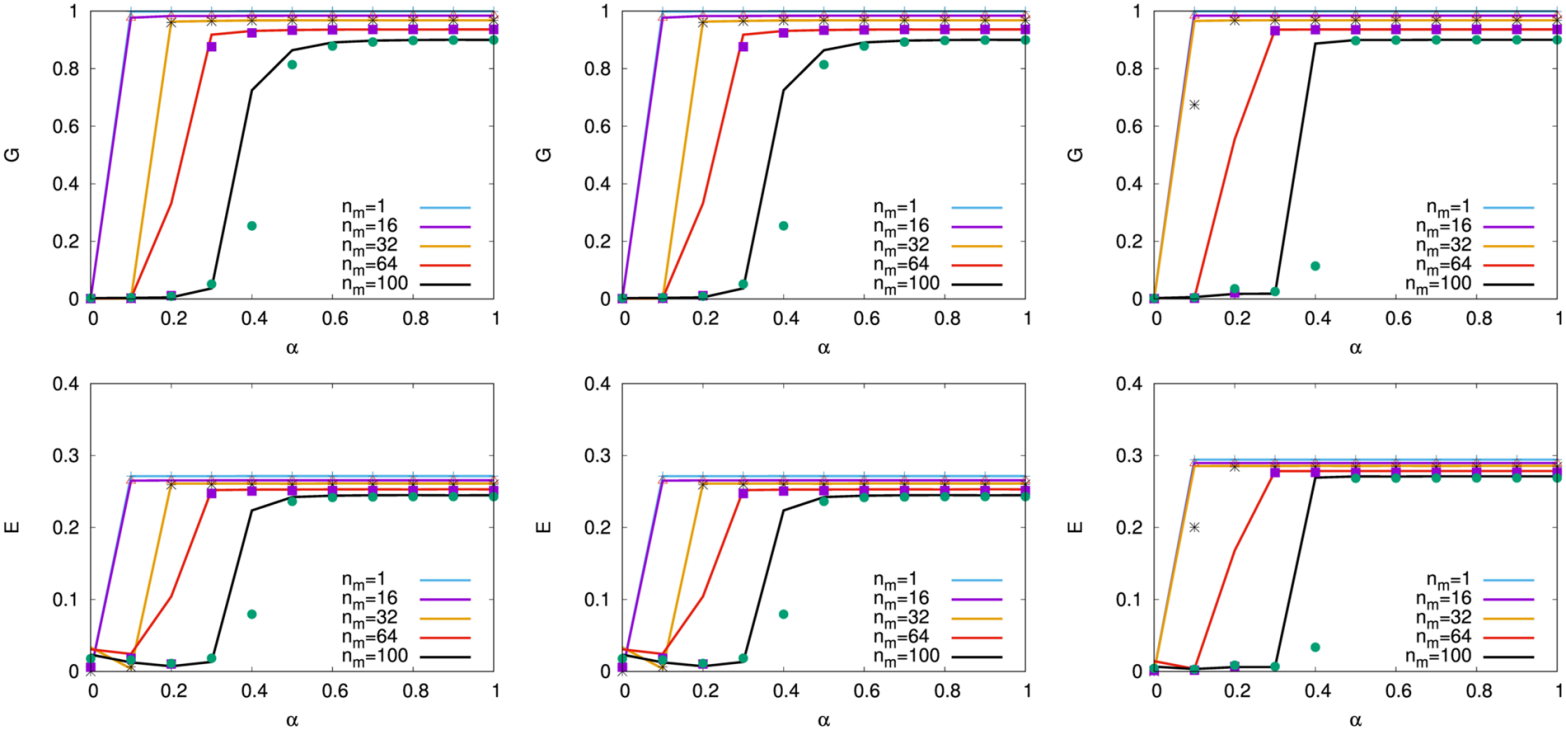
Tolerance against the trigger of multi-attacks for our detour routing on (Left) SF networks, onion-like networks by MED-kmin of (Middle) *μ* = 0, and (Right) *μ* = 4. Line and mark distinguish the cases of simultaneously removing *n*_*m*_ nodes selected in decreasing order of degree and load from the maximum, respectively, however there is little difference between them.

Instead of the initial attack to a node, we study the tolerance against multiple targeted attacks^23,24^ by simultaneously removing *n*_*m*_ nodes selected in decreasing order of degree or load as the trigger in the total 10^3^ nodes, since these nodes are considered as the weakest parts for cascading failures. As shown in the left and right of Fig. 9, SF networks are not sustainable with high *G* and *E* from *n*_*m*_ = 8,16 (blue and purple lines): 1% node removals of trigger around *α* ≈ 0.5 (as a reasonable setting: *C*_*i*_ is 1.5 times larger than the initial load in Eq. (1)), while onion-like networks have strong tolerance even for *n*_*m*_ = 64,100 (red and black line): 10% node removals of trigger. The orange, red, black lines for *n*_*m*_ = 32,64,100 in the middle of Fig. 9 show that for *α* < 0.5 the tolerance in onion-like networks by MED-kmin of *μ* = 0 becomes somewhat weaker than that in the right of Fig. 9. This degradation is consistent with the result of low efficiency *E* in the right of Fig. 8.

## Discussion

We have proposed a pair of attachments for incrementally growing onion-like networks with positive degree-degree correlations^2,3^, and shown a further improvement of robustness against the intelligent HDA and BP attacks^8,9^ than the previous method^6^. One of the attachments is based on uniformly random selection, and contributes to enhancing the correlations among large degree nodes. Another is based on the selection of minimum degree node in the neighbors of a few hops through range-limited intermediations from the the randomly chosen pair node, and contributes to enhancing the correlations among small degree nodes. We have numerically investigated that the enhancement of interwoven loops by increasing the size of FVS is crucial to the improvement of robustness in focusing on design principle beyond process level of rewiring^7^. Moreover, we have found out that onion-like networks acquire adaptive capacity in resilience^13^ by a change of policy for flow control from usual shortest-based to congestion-aware load-based routing in order to absorb cascading overload failures triggered by malicious attacks, and that our congestion-aware detour strategy is superior to both the defense^14^ and the navigation^15^ strategies. In particular, onion-like networks with bypasses originated from loops have strong tolerance against trigger of multi-attacks in comparison with SF networks found in many real systems. As one of unsolved subjects, it is an important issue to understand complex behavior and predict it in multi-scale (or multilayer, interdependent) networks of techno-social systems^25^. For cascading failures, some studies^26–28^ have made a challenge to the problems on interdependent networks which are beyond our current scope. In addition, as other strategies to be resilient networks, repairing or healing^29–31^ by adding rewired links can be considered, however they remain in future studies.

Supplementary, we discuss an explanation from organization theory. The connections between randomly chosen and the distant nodes via new node in our proposed networks correspond to *long-distance relations* in case studies in organization theory: long-distance relations led to overcome the crisis of Toyota group’s supply chain damaged by large fire accident to their subcontract plants^32–34^. Usual connections among different suppliers through voluntary meetings in the intentional Toyota’s strategy quickly reconstruct other productions by using the intermediations based on trust rather than immediate profit in building long-term win-win relations. The power of complex web of Toyota’s communication networks has been pointed out as follows^33,35^.

- *As a result of these relations, employees at Toyota belong to large numbers of committees (iinkai), self-organizing study groups (jishuken), and informal groups*.
- *The internal structure of Toyota support the free exchange of ideas, emphasizing the communications of differences to improve operations and resolve problems*.
- *Operating on the assumption that “everybody knows everything,” information within Toyota flows freely up and down the hierarchy and cross functional and seniority levels, extending outside the organization to suppliers, customers, and dealers*.

The long-distance relations are also useful for rapidly organizing world-wide economic networks with expanding business chances by Wenzhou people in China^34^. More than 400,000 Wenzhou people go out abroad, and the half prospers by making business networks for daily necessary garments or leathers in Europe. By entrusting something in cooperation with each other, intermediations of human, goods, and funds bridge structural holes^36^ between lingual, cultural, organizational, or geological gaps including their home-town and the distant partner’s places located in world-wide.

On the brain circulation system known as Silicon Valley (SV) model, by immigrant engineers, shortcut connections between SV and his/her home country such as China or India strongly contribute for developing innovational high-tech industry with market opportunities^37^. The established connections via intermediations probably work well for managing cross-border operations. These case studies suggest the universal importance of long-distance relations through daily cooperation for enhancing both robustness and efficiency of network. From mere relations, by our proposed growing methods, the importance is extended to interwoven loops for constructing onion-like networks which give the strongest robustness in the state-of-the-art network science. It is an issue for future infrastructure of socio-technological systems how such intermediations will be able to be naturally realized beyond selfish thinking of preferential attachment^16^ in growing organizational networks including communications or transportations.

## Methods

### Approximation method for finding FVS

We briefly review an approximation method for finding FVS. This method is not our original. As mentioned in ref.^17^, it is assumed that nodes *j* ∈ ∂*i* are mutually independent of each other when node *i* is removed. Here, ∂*i* denotes the set of connecting neighbor nodes of *i*. Such approximated tree-like graph is called cavity graph in statistical physics. Let us consider the marginal probability ${q}_{i}^{{A}_{i}}$ for the state *A*_*i*_ of node *i*. Since *A*_*i*_ represent the index of root node of *i*, it is influenced by the neighbor nodes in the cavity graph after removing node *i* denoted by \*i*. Based on the product of independent marginal probability ${q}_{j\to i}^{{A}_{j}}$ for the state *A*_*j*_, the joint probability is

$${{\mathscr{P}}}_{\backslash i}({A}_{j}:j\in \partial i)\approx {{\rm{\Pi }}}_{j\in \partial i}{q}_{j\to i}^{{A}_{j}}\mathrm{.}$$

In the cavity graph, if all nodes *j* ∈ ∂*i* are either empty (*A*_*j*_ = 0) or roots (*A*_*j*_ = *j*), the added node *i* can be a root (*A*_*i*_ = *i*). There are the following exclusive states.

1. *A*_*i*_ = 0: *i* is empty (removed). Since *i* is unnecessary as a root, it belongs to FVS.
2. *A*_*i*_ = *i*: *i* becomes its own root.
  The state *A*_*j*_ = *j* of *j* ∈ ∂*i* is changeable to *A*_*j*_ = *i* when node *i* is added.
3. *A*_*i*_ = *k*: one node *k* ∈ ∂*i* becomes the root of *i* when it is added, if *k* is occupied and all other *j* ∈ ∂*i* are either empty or roots.

The corresponding probabilities to the above three states are represented by

2 $$\begin{array}{lll}{q}_{i}^{0} & \mathop{=}\limits^{{\rm{def}}} & \frac{1}{{z}_{i}(t)},\\ {q}_{i}^{i} & \mathop{=}\limits^{{\rm{def}}} & \frac{{e}^{x}{{\rm{\Pi }}}_{j\in \partial i(t)}\,[{q}_{j\to i}^{0}+{q}_{j\to i}^{j}]}{{z}_{i}(t)},\\ {q}_{i}^{k} & \mathop{=}\limits^{{\rm{def}}} & \frac{{e}^{x}\frac{\mathrm{(1}-{q}_{k\to i}^{0})}{{q}_{k\to i}^{0}+{q}_{k\to i}^{k}}{{\rm{\Pi }}}_{j\in \partial i(t)}\,[{q}_{j\to i}^{0}+{q}_{j\to i}^{j}]}{{z}_{i}(t)},\end{array}$$

3 $${q}_{i\to j}^{0}=\frac{1}{{z}_{i\to j}(t)},$$

4 $${q}_{i\to j}^{i}=\frac{{e}^{x}{{\rm{\Pi }}}_{k\in \partial i(t)\backslash j}[{q}_{k\to i}^{0}+{q}_{k\to i}^{k}]}{{z}_{i\to j}(t)},$$

where ∂*i*(*t*) denotes node *i*'s set of connecting neighbor nodes at time *t*, and *x* > 0 is a parameter of inverse temperature. We have the normalization constant

5 $${z}_{i}(t)\mathop{=}\limits^{{\rm{def}}}1+{e}^{x}[1+\sum _{k\in \partial i(t)}\frac{1-{q}_{k\to i}^{0}}{{q}_{k\to i}^{0}+{q}_{k\to i}^{k}}]{{\rm{\Pi }}}_{j\in \partial i(t)}[{q}_{j\to i}^{0}+{q}_{j\to i}^{j}],$$

6 $${z}_{i\to j}(t)\mathop{=}\limits^{{\rm{def}}}1+{e}^{x}{{\rm{\Pi }}}_{k\in \partial i(t)\backslash j}[{q}_{k\to i}^{0}+{q}_{k\to i}^{k}]\times [1+\sum _{l\in \partial i(t)\backslash j}\frac{1-{q}_{l\to i}^{0}}{{q}_{l\to i}^{0}+{q}_{l\to i}^{l}}],$$

to be satisfied for any *i* and *i* → *j* as

$${q}_{i}^{0}+{q}_{i}^{i}+\sum _{k\in \partial i}{q}_{i}^{k}=\mathrm{1,}$$

$${q}_{i\to j}^{0}+{q}_{i\to j}^{i}+\sum _{k\in \partial i}{q}_{i\to j}^{k}=1.$$

The massage-passing iterated by Eqs (2–6) is called belief propagation (BP). This calculation of ${q}_{i}^{0}$, ${q}_{i}^{i}$, ${q}_{i}^{k}$, ${q}_{i\to j}^{0}$, ${q}_{i\to j}^{i}$, and ${q}_{i\to j}^{k}$ is executed through the massage-passing until to be self-consistent in principle but practically to reach appropriate rounds from initial setting of (0, 1) random values. The unit time from *t* to *t* + 1 for calculating a set $\{{q}_{i}^{0}\}$ consists of a number of rounds by the updating Eqs (2–6) in order of random permutation of *N* nodes. Thus, as a candidate of FVS^17^ or the target of BP attack^9^, a node of the highest ${q}_{i}^{0}$ is chosen and removed with recalculation for the remaining subgraph at *t*. Since the precise process in BP attack stops after all loops have been destroyed^9^, we continue it for the component of remaining trees by switching to HDA attack until removing nodes of fraction *q*.

### Strategies against cascading overload failures

We explain the conventional defense and navigation strategies. A defense strategy based on intentional removal of nodes has been proposed^14^. As sacrifices, the fraction *f*_*s*_ of nodes with the smallest ${{\rm{\Delta }}}_{i}\mathop{=}\limits^{{\rm{def}}}{L}_{i}\mathrm{(0)}-{L}_{i}^{g}$ are intentionally removed to avoid the heavy generation of packets from the peripheral nodes that rarely contribute to transmitting packets. The total load generated by node *i* is

$${L}_{i}^{g}\mathop{=}\limits^{{\rm{def}}}\sum _{j\ne i}({D}_{ij}+\mathrm{1).}$$

The remaining quantity Δ_*i*_ from the initial load *L*_*i*_(0) is the part that contributes to transmitting packets at node *i* before the trigger of attack. In this defense strategy, after removing the sacrificed nodes, cascading process is executed in updating loads {*L*_*i*_(*t*)} based on the shortest paths without a change of routing policy.

A navigation strategy has been also proposed^15^ in considering a combination of shortest and degree-based paths. For any path *P*(*i* → *j*) through *i* = *v*_0_, *v*_1_, …, *v*_*n*−1_, *v*_*n*_ = *j*, the efficient path that minimizes the weighted length

$$L(P(i\to j):w)\mathop{=}\limits^{{\rm{def}}}\sum _{i=1}^{n-1}(1-w+w\times \frac{k({v}_{i})}{{k}_{max}}),$$

is selected. Here, *k*(*v*_*i*_) denotes the degree of node *v*_*i*_, *k*_*max*_ is the largest degree in the network, and 0 ≤ *w* ≤ 1 is a weight parameter. In particular, when *w* = 0, *L*(*P*(*i* → *j*):0) corresponds to the traditional shortest path, when *w* = 1, *L*(*P*(*i* → *j*):1) corresponds to the degree-based path which avoids passing through large degree nodes usually with much load. Apart from the shortest-paths, we chose *w* = 0.5 to avoid the decreasing of *G* in *w* ≈ 1.0. The efficient paths are found for all combinations of source and terminal in *N* nodes in one unit time. Note that the ordering of selection of source and terminal nodes does not affect the determination of paths that minimize *L*(*P*(*i* → *j*): *w*). In cascading failures after the trigger, at every time step *t* ≥ 1, the loads {*L*_*i*_(*t*)} are updated in a similar way to the betweenness centrality on the efficient path instead of the shortest path.

On the other hand, we propose a congestion-aware routing with random order of transfers between two nodes *i* and *j* in order to drastically reduce cascading overload failures. In our detour strategy, we chose a path that minimizes the sum of one hop and fraction of load defined by

7 $$1+\frac{{L}_{{v}_{k}}(\tau )}{{C}_{{v}_{k}}},$$

for connecting nodes *i* = *v*_0_, *v*_1_, …, *v*_*k*_, …, *v*_*n*−1_, *v*_*n*_ = *j*. By the second term of penalty in Eq. (7), this path tends to avoid the passing through congested nodes with much load at *τ*, and to distribute packet flows. In randomly selected order of source and terminal nodes *i* and *j*, the detour paths are found by minimizing the sum of Eq. (7) for connecting nodes in the interval Δ*τ* = unit time × 2/*N*(*N* − 1). After finding the detour paths from *i* to *j*, the load at *v*_1_, …, *v*_*k*_, …, *v*_*n*−1_ on the paths is updated by

8 $${L}_{{v}_{k}}(\tau +{\rm{\Delta }}\tau )\leftarrow {L}_{{v}_{k}}(\tau )+\frac{{\sigma }_{ij}({v}_{k})}{{\sigma }_{ij}},$$

where *σ*_*ij*_ denotes the number of detour paths and *σ*_*ij*_(*v*_*k*_) is the number of paths as the subset that is passing through node *v*_*k*_ in the detour paths. The routing process according to Eqs (7,8) is repeated for the next source and terminal nodes at *τ* ← *τ* + Δ*τ*. In one unit time, all paths for the combination of *N* nodes as source and terminal are found. Although we can consider the weighted version 1 − *w* + *wL*_*i*_(*t*)/*C*_*i*_, it has same results in the resilience of network for 0.01 ≤ *w*< 1.0 (see Supplementary information, S2). Note that the case of *w* → 0 is corresponded to the shortest-based routing.

## Electronic supplementary material

Supplementary Information
