# Author Correction: Onion-like networks are both robust and resilient

**DOI:** 10.1038/s41598-018-32563-3

**Published:** 2018-09-26

**Authors:** Yukio Hayashi, Naoya Uchiyama

**Affiliations:** Japan Advanced Institute of Science and Technology, Graduate School of Advanced Institute of Science and Technology/Division of Transdisiplinary Sciences, Ishikawa, 923-1292 Japan

Correction to: *Scientific Reports* 10.1038/s41598-018-29626-w, published online 26 July 2018

In Figure 9, the graphs on the Left are incorrect duplications of the graphs in the Middle, in both rows. The correct Figure 9 appears below as Figure 1.

**Figure 1:**
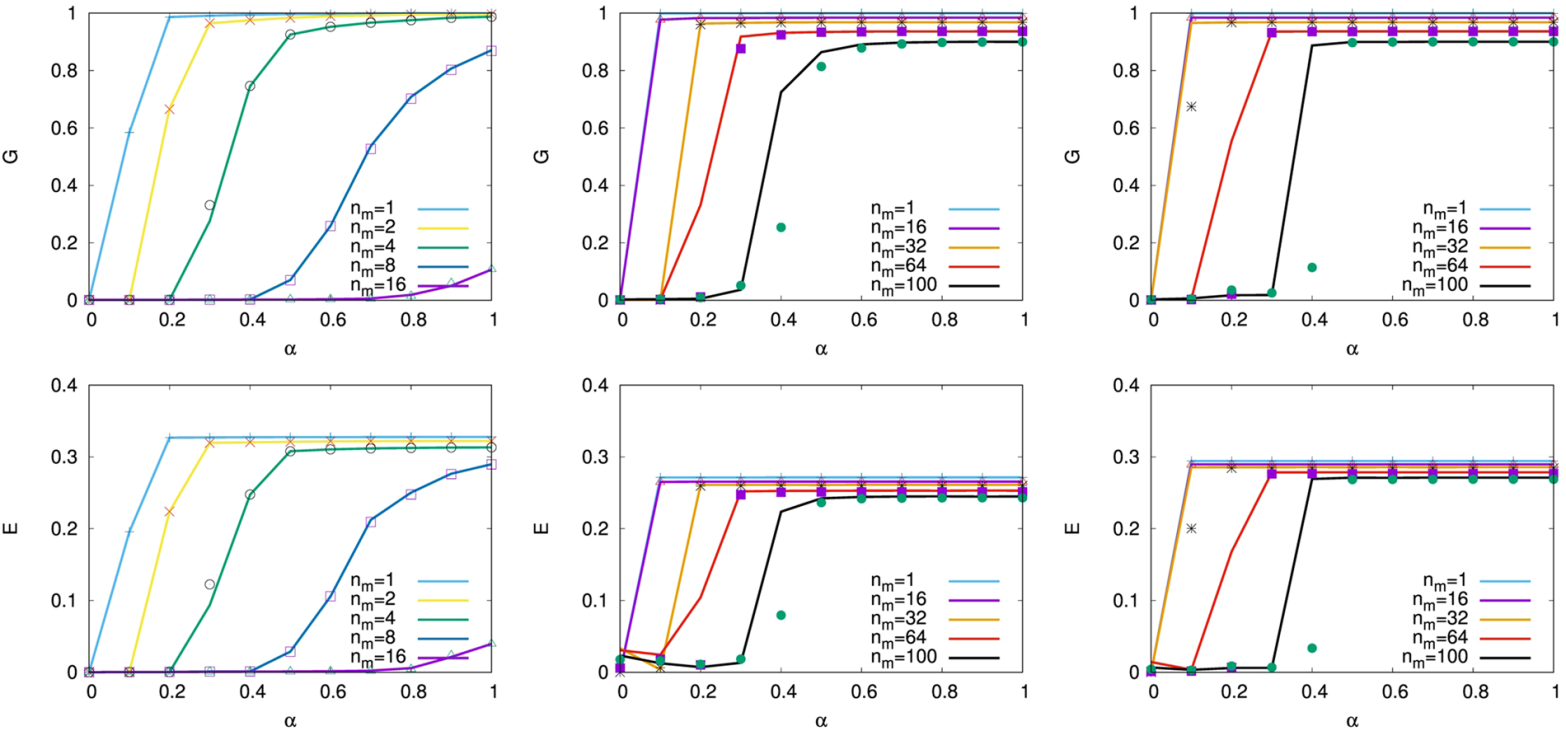
Tolerance against the trigger of multi-attacks for our detour routing on (Left) SF networks, onionlike networks by MED-kmin of (Middle) *μ* = 0, and (Right) *μ* = 4. Line and mark distinguish the cases of simultaneously removing *n*_*m*_ nodes selected in decreasing order of degree and load from the maximum, respectively, however there is little difference between them.

