# Supplementary material for: Onion-like networks are both robust and resilient
Source: Sci Rep. 2018 Jul 26;8:11241. doi: 10.1038/s41598-018-29626-w (PMC6062544; doi:10.1038/s41598-018-29626-w)
Supplement: Supplementary file 1 — Supplementary Information [file 41598_2018_29626_MOESM1_ESM.pdf]

SI for “Onion-like networks are both robust and resilient”

Yukio Hayashi[1,*] **and Naoya Uchiyama**[1]

[1]Japan Advanced Institute of Science and Technology, Graduate School of Advanced Institute of Science and Technology/ Division of Transdisiplinary Sciences, Ishikawa, 923-1292, Japan

ABSTRACT

This is the supplementary Information (SI) for “Onion-like networks are both robust and resilient”.

S 1. Strong correration of R and $|FVS|$

The following table shows the strong correlation coefficient of the robustness index R_{hub} or R_{bp} and the size of FVS in growing onion-like networks until $N = 5000$ by MED-kmin with $\mu = 0, 1, 2, 3, 4$.

μ	R_{hub}&$\|FVS\|$	R_{hub}&$\|FVS\|$
0	0.7926	0.8213
1	0.7381	0.7789
2	0.7381	0.7898
3	0.8294	0.8673
4	0.9306	0.9467

Figure S1 shows the fraction of the size of FVS in growing onion-like networks from each initial configuration of a)-d). The order of lines from top to bottom is corresponding to decreasing order of the robustness against the worst BP attacks in Inset of Fig. 5.

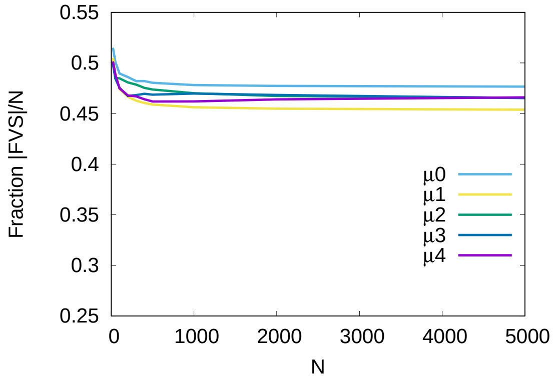

a) Initial Complete:K_5

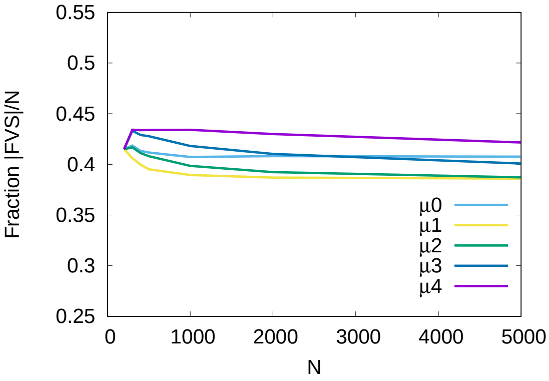

b) Initial ER:Poisson

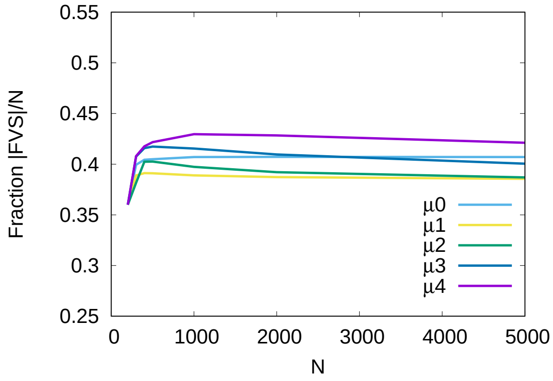

c) Initial Exp.Dist.

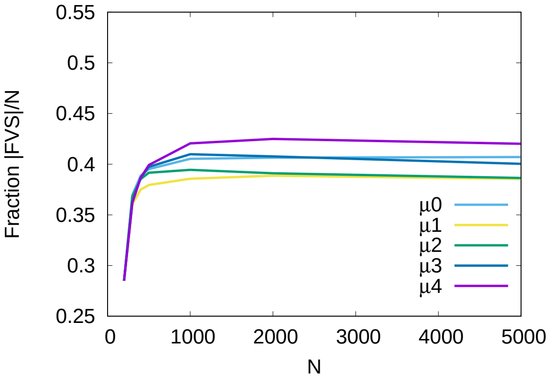

d) Initial SF by BA model

Figure S1. Fraction of the size of FVS in growing onion-like networks. These results are averaged over 100 realizations.

S 2. Weighted version of our detour routing in cascading failures

As shown in Fig. S2, for the weighted version

$$(1-w)+w\times\frac{L_i(\tau)}{C_i},$$

we have the same result to the original in Eq.(7) at the case $w=0.5$.

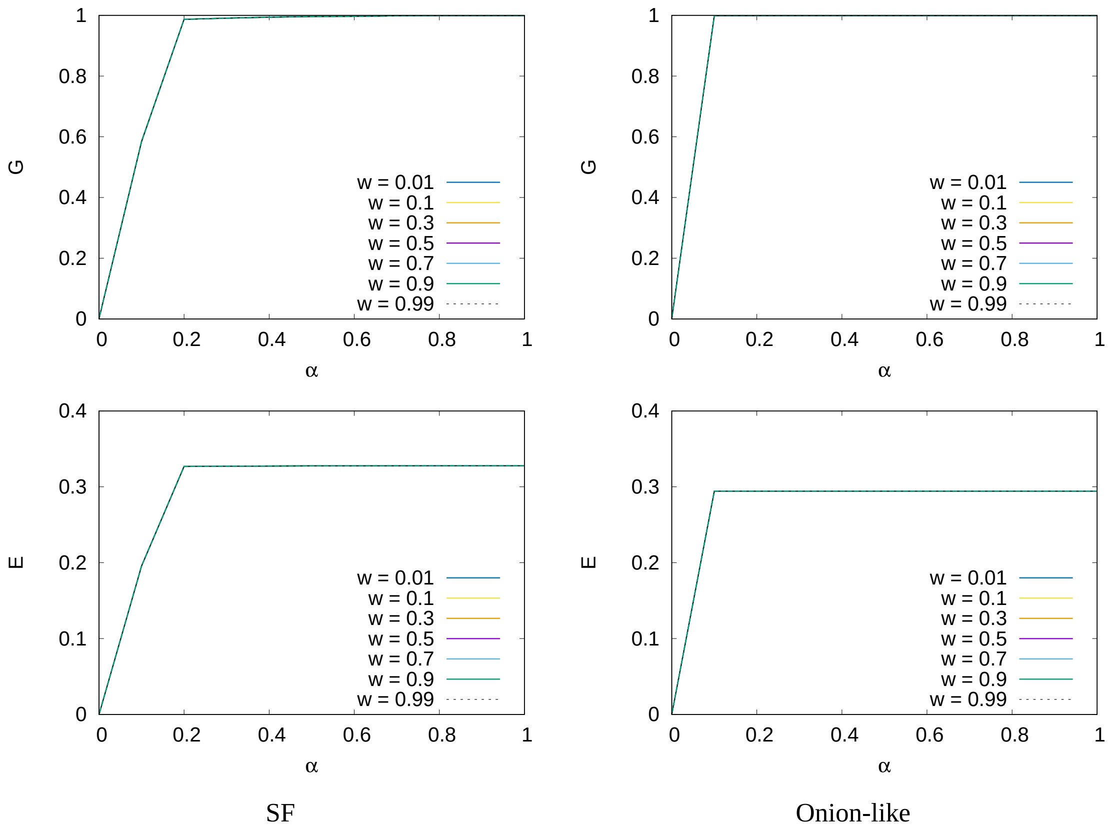

Figure S2. Coincidence of lines for varing the wight w. These results are averaged over 10 realizations

S 3. Detailed results for other setteing of capacity in cascading failure

We consider other setteing of capacity:

- Dou et al.'s setting

$$C_i \stackrel{\text{def}}{=} L_i(0) + \alpha' L_i(0)^{\beta},$$

- Zhao et al.'s setting

$$C_i \stackrel{\text{def}}{=} \left(1 + \alpha' \left(\frac{k_i}{k_{max}}\right)^{\beta}\right) L_i(0),$$

where α' is set as $\alpha \sum_i L_i(0) / \sum_i L_i(0)^{\beta}$ and $\alpha \sum_i L_i(0) / \sum_i (k_i/k_{max})^{\beta} L_i(0)$, respectively, for $0 \leq \alpha \leq 1$ and $0.2 \leq \beta \leq 1.4$ in order to be equivalent to the total capacity of load $\sum_i C_i$ defined by Eq.(1). Note that tha case of $\beta = 1.0$ in Dou et al.'s setting is equivalent to the original of Eq.(1).

We obtain the advantage of our routing as follows.

- Figures S3 $\sim$ 9 show the surviving size G of giant component and network efficiency E by conventional and our routing strategies for the tolerant parameter α in cascading failures on SF networks by BA model and onion-like networks by MED $\mu = 4$.
- Figures S10 $\sim$ 13 are the rearrange to compare them for the value of β in SF networks.
- Figures S14 $\sim$ 17 are the rearrange to compare them for the value of β in onion-like networks.
- Figures S18, 19 show the comparison for the value of β in the results by our routing.

These results are averaged over 10 realizations

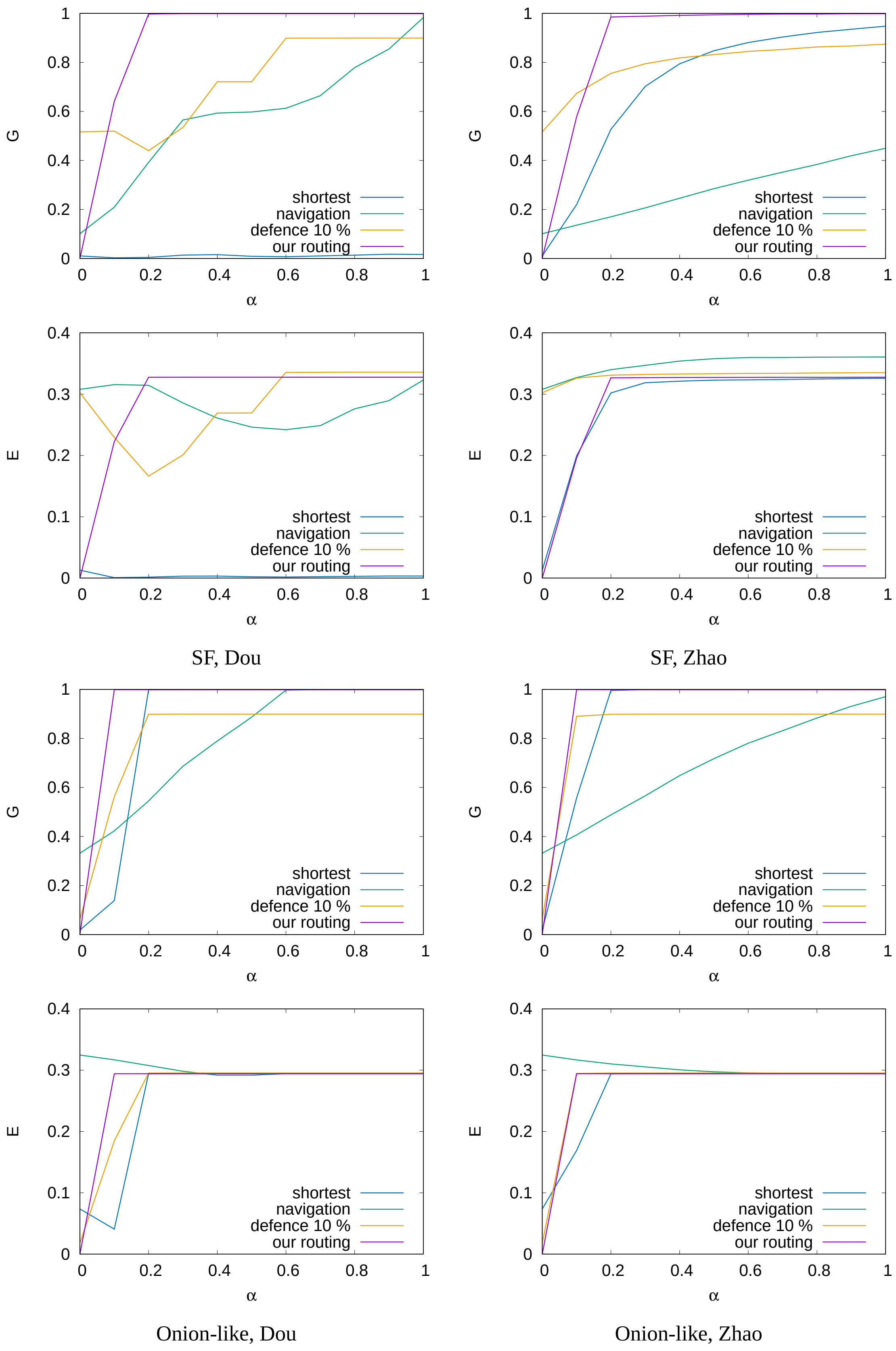

Figure S3. $\beta = 0.2$, (top) SF, (bottom) onion-like networks $\times$ (left) Dou's, (right) Zhao's settigs of C_i.

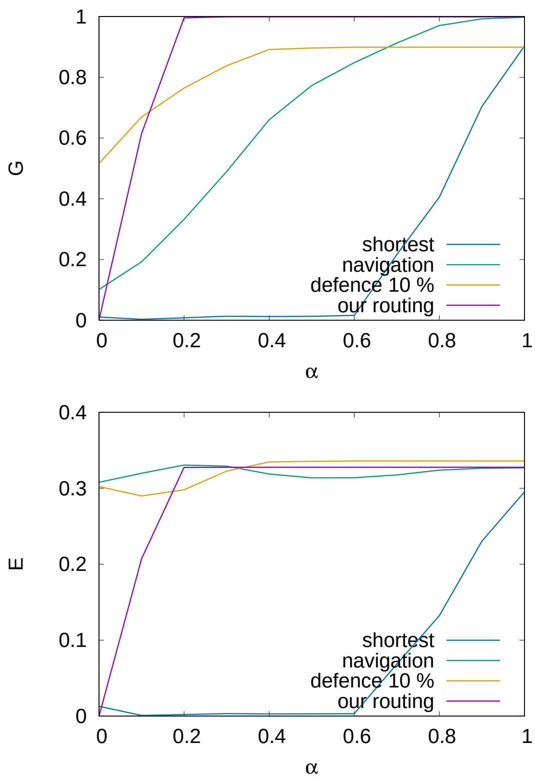

SF, Dou

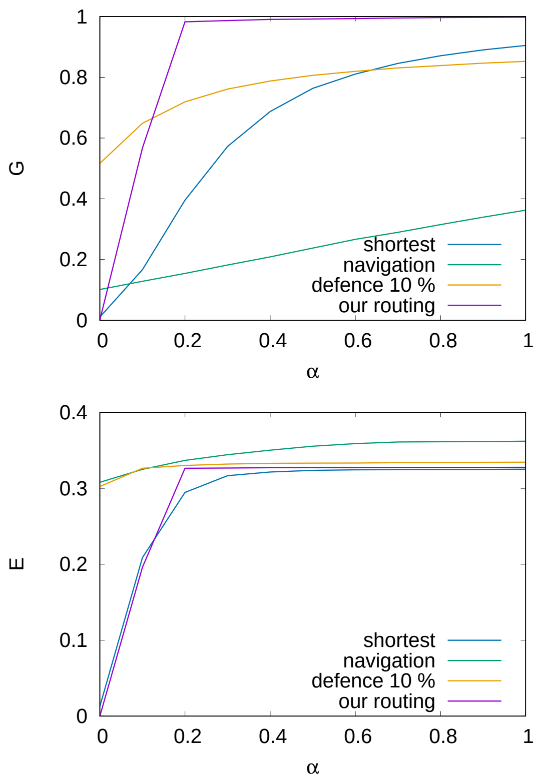

SF, Zhao

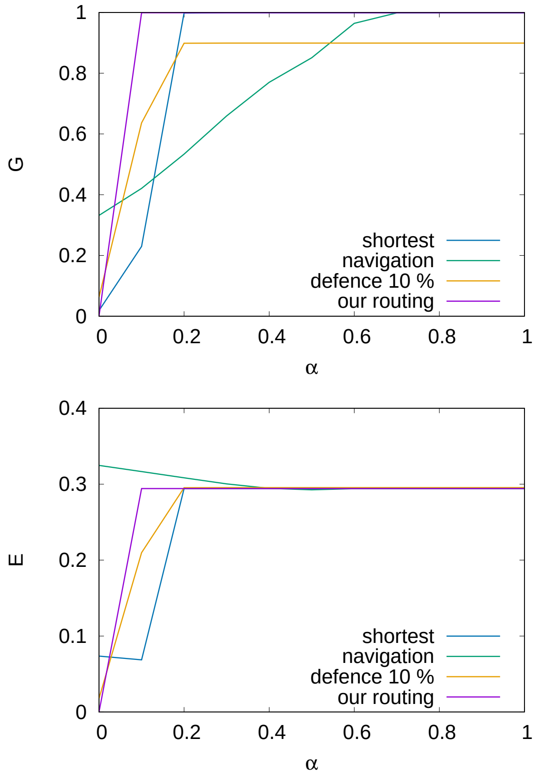

Onion-like, Dou

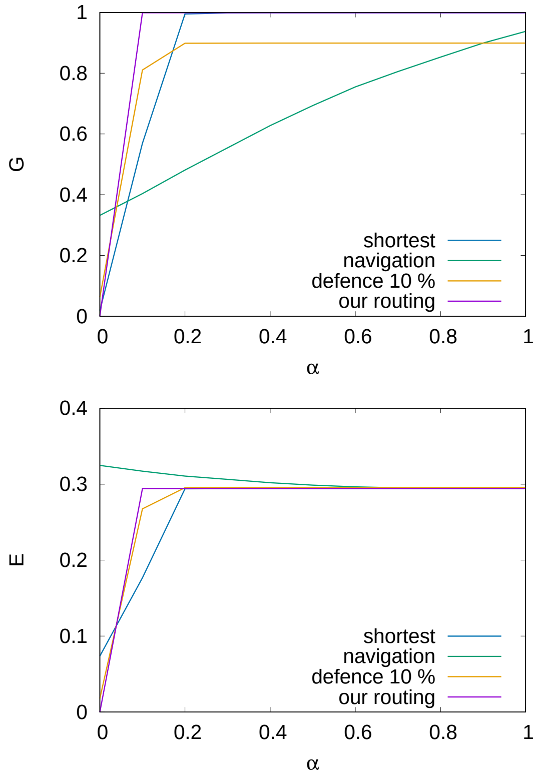

Onion-like, Zhao

Figure S4. $\beta = 0.4$, (top) SF, (bottom) onion-like networks $\times$ (left) Dou's, (right) Zhao's settigs of C_i.

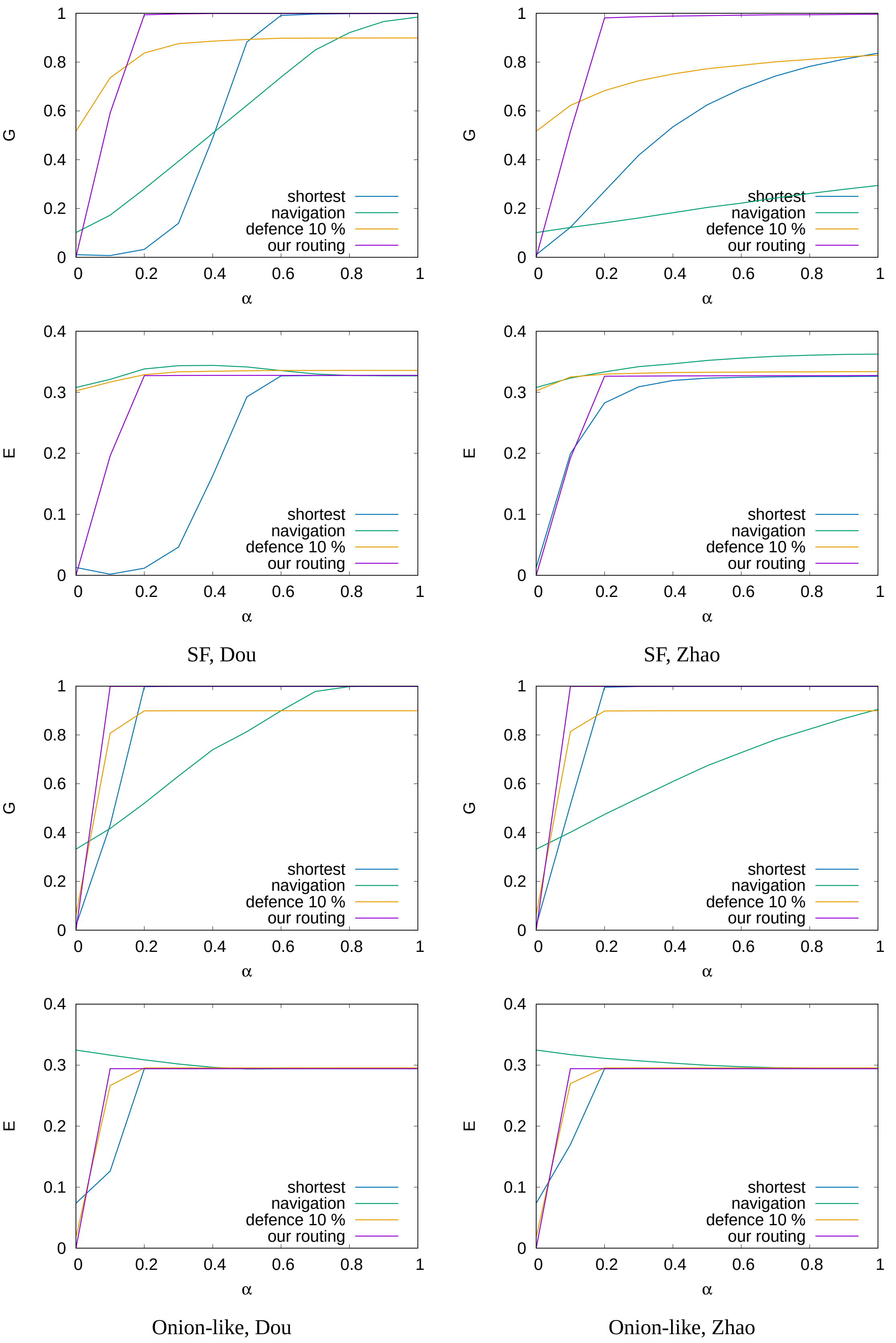

Figure S5. $\beta = 0.6$, (top) SF, (bottom) onion-like networks $\times$ (left) Dou's, (right) Zhao's settigs of C_i.

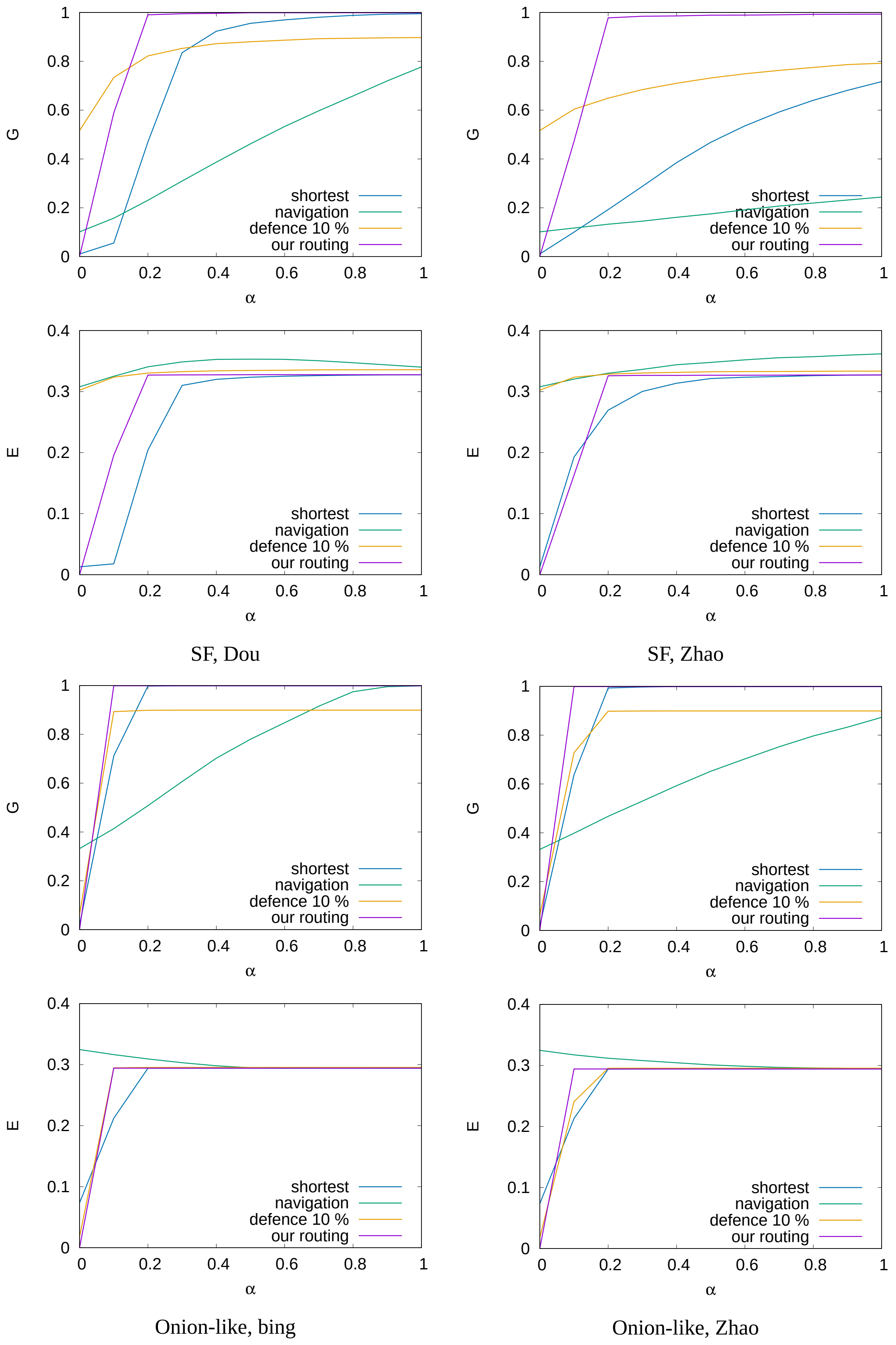

Figure S6. $\beta = 0.8$, (top) SF, (bottom) onion-like networks $\times$ (left) Dou's, (right) Zhao's settigs of C_i.

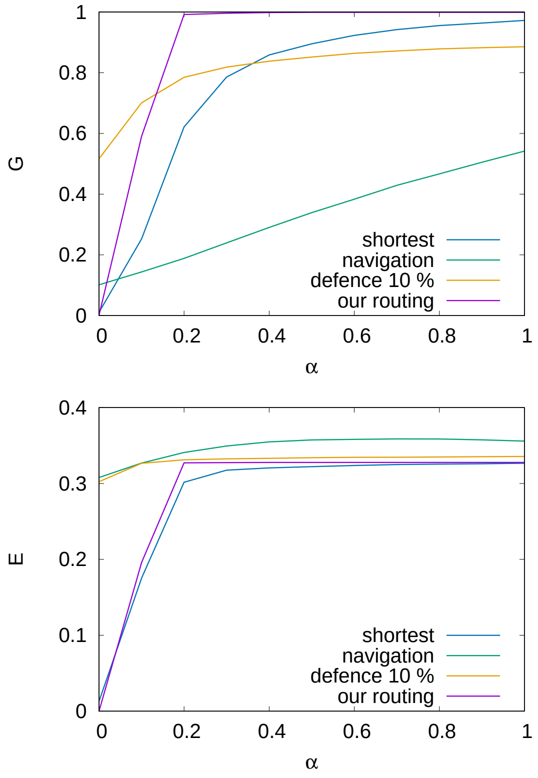

SF, Dou

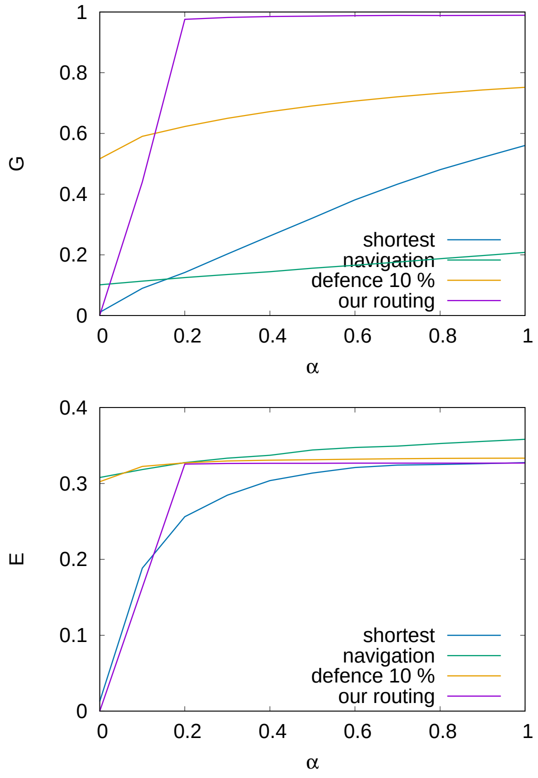

SF, Zhao

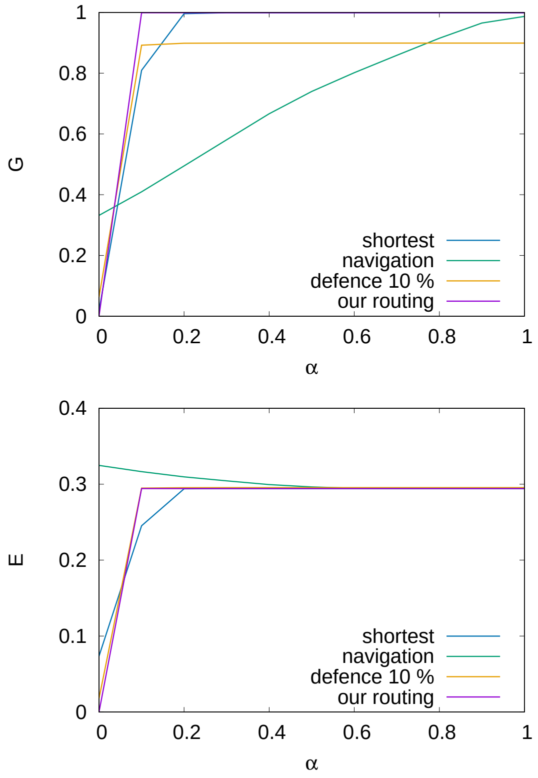

Onion-like, Dou

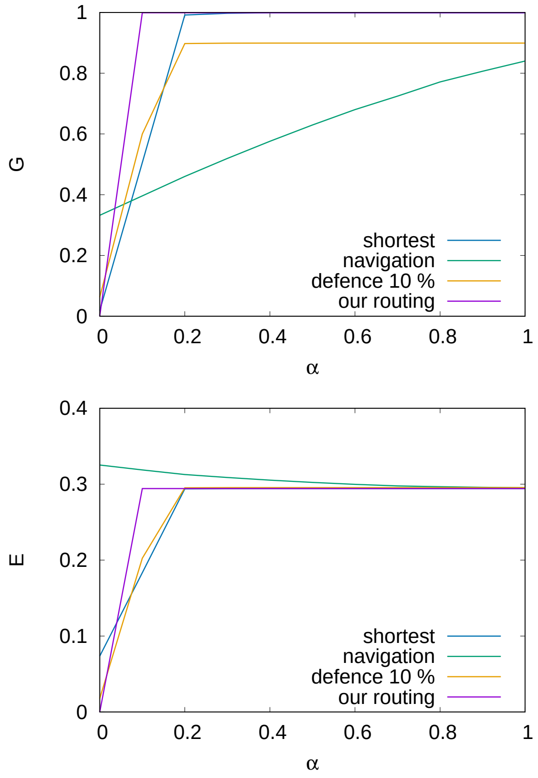

Onion-like, Zhao

Figure S7. $\beta = 1.0$, (top) SF, (bottom) onion-like networks $\times$ (left) Dou's, (right) Zhao's settigs of C_i.

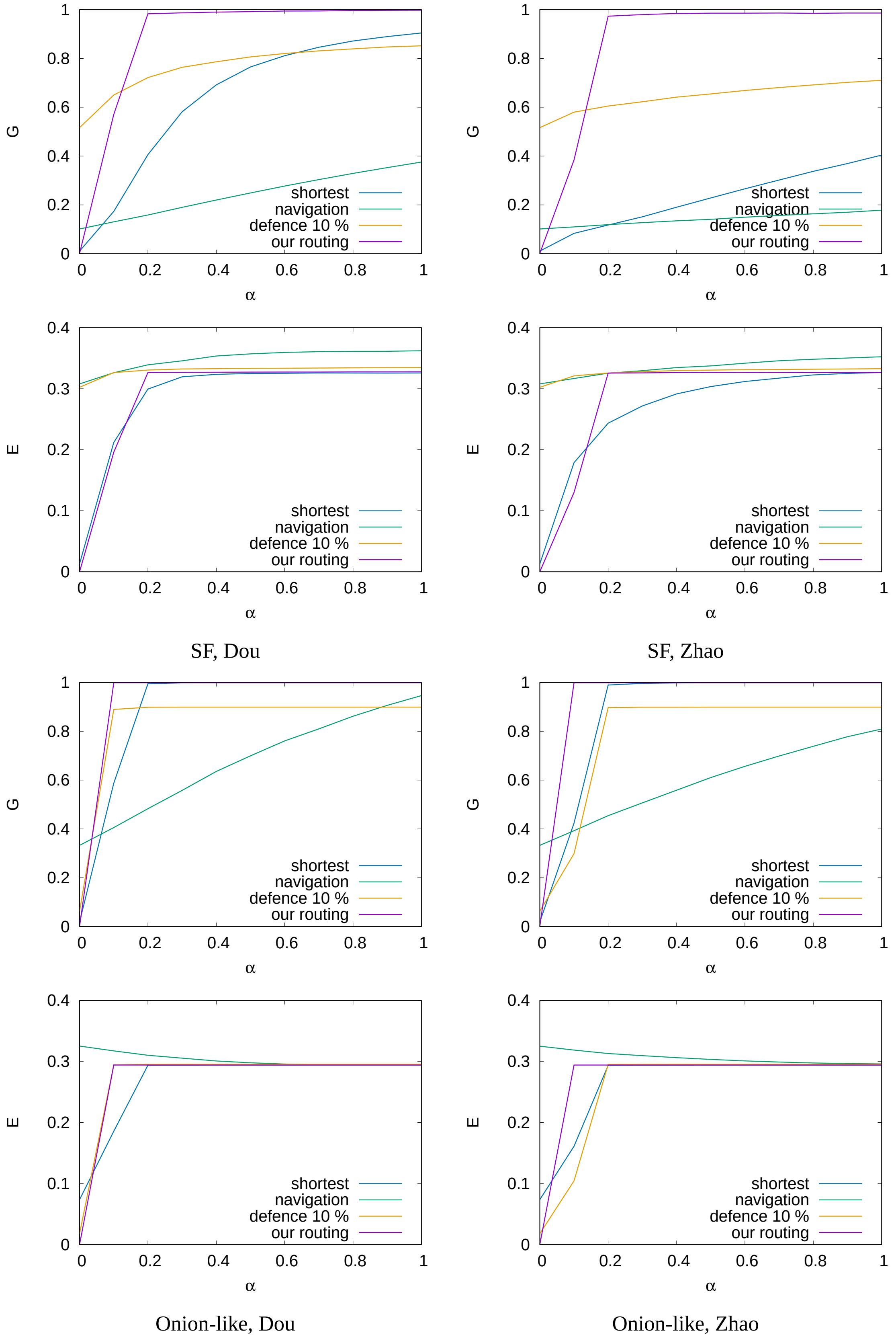

Figure S8. $\beta = 1.2$, (top) SF, (bottom) onion-like networks $\times$ (left) Dou's, (right) Zhao's settigs of C_i.

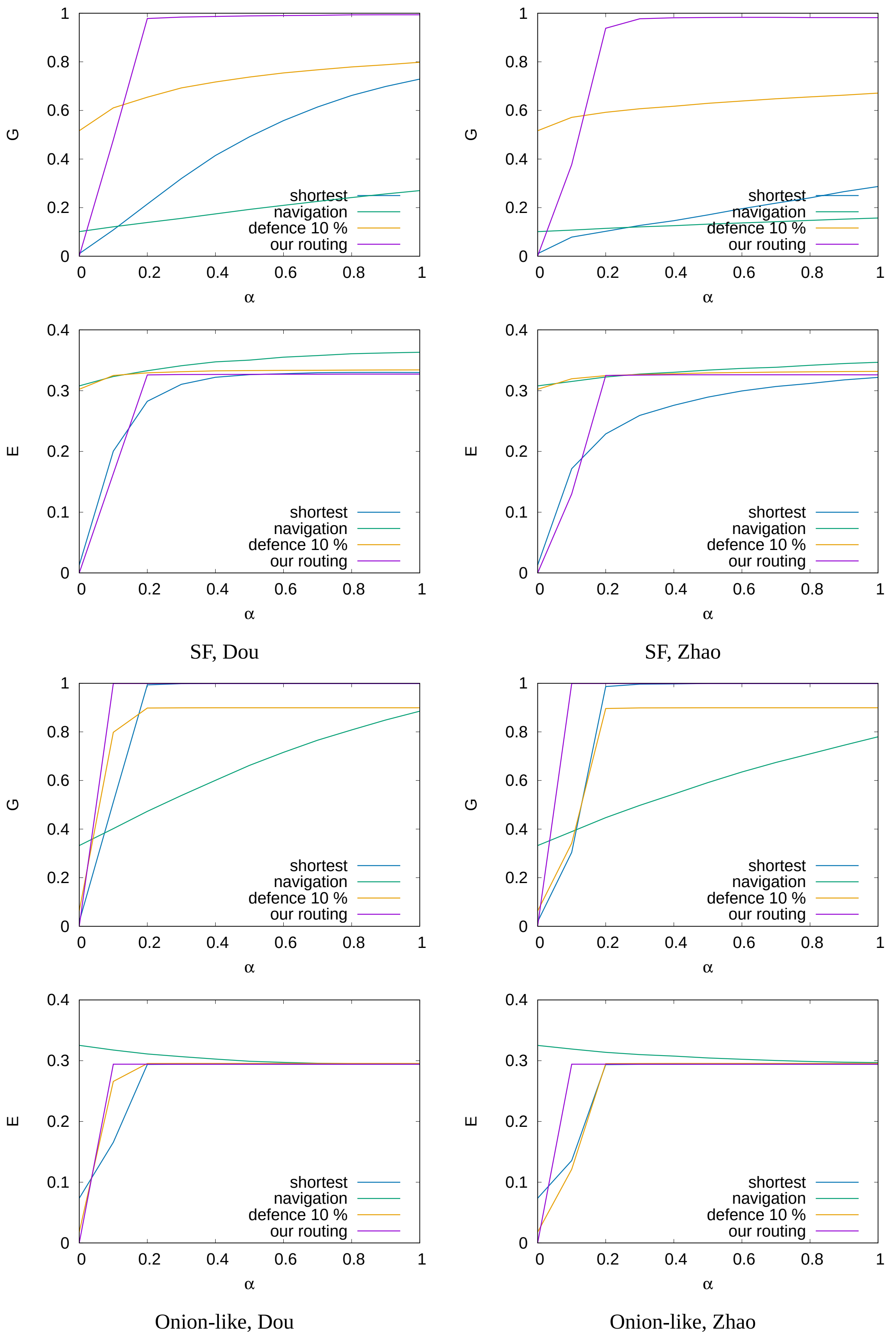

Figure S9. $\beta = 1.4$, (top) SF, (bottom) onion-like networks $\times$ (left) Dou's, (right) Zhao's settigs of C_i.

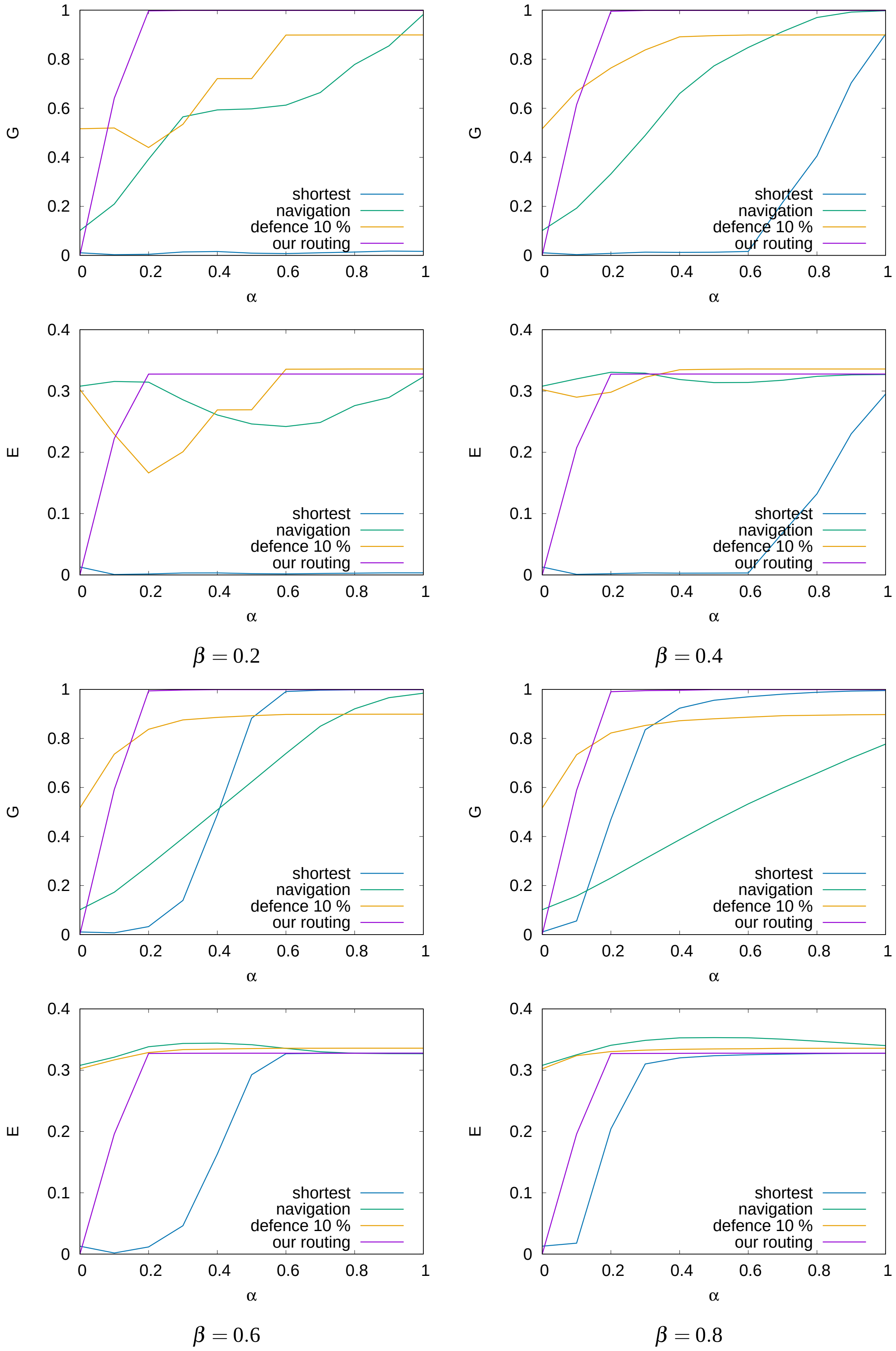

Figure S10. Comparison for the value of β in SF networks, Dou's settig.

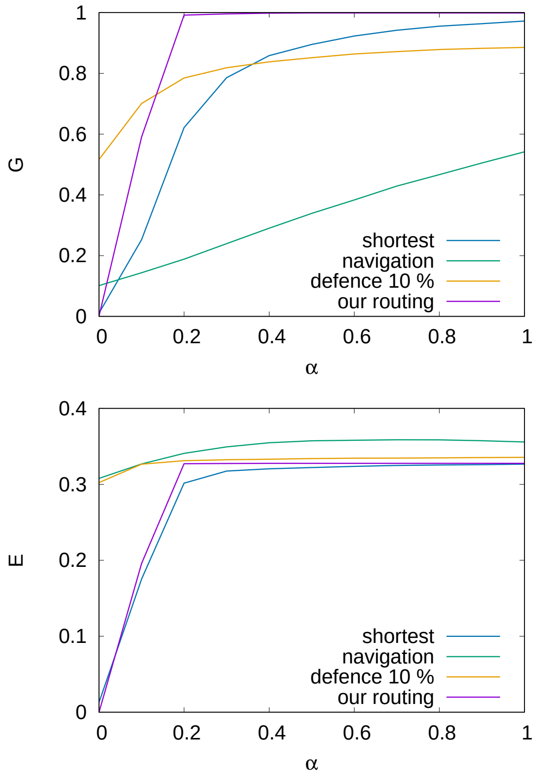

$\beta = 1.0$

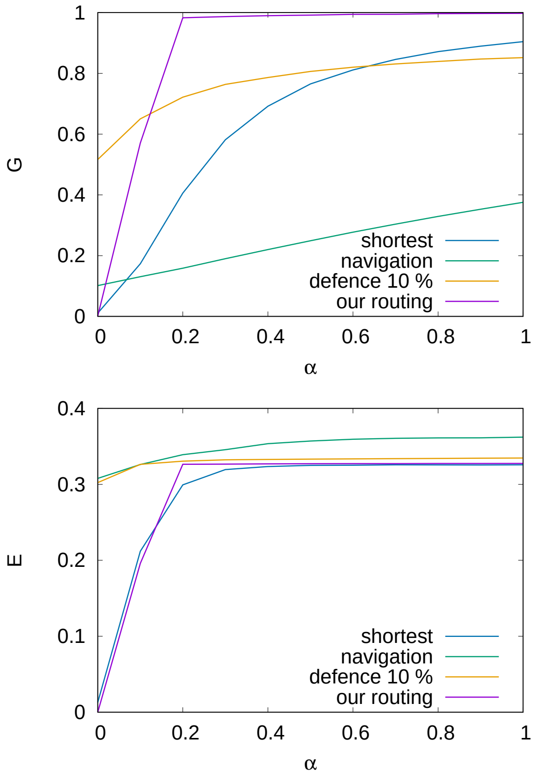

$\beta = 1.2$

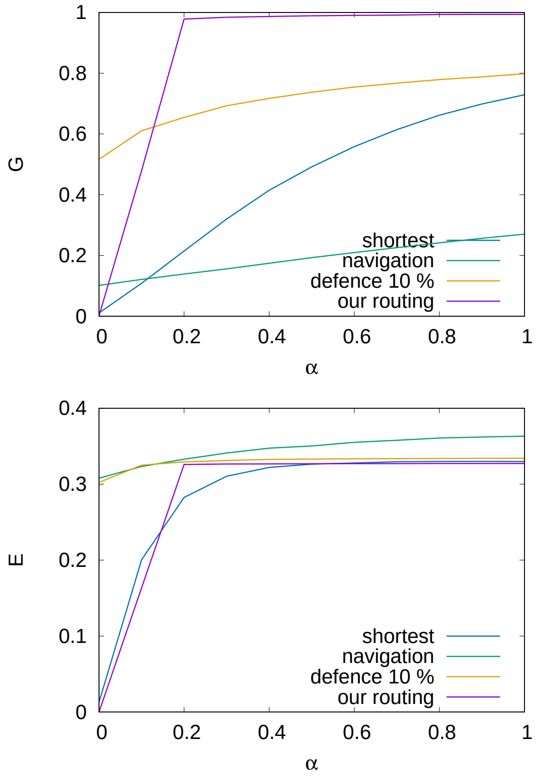

$\beta = 1.4$

Figure S11. (Continue) Comparison for the value of β in SF networks, Dou's settig.

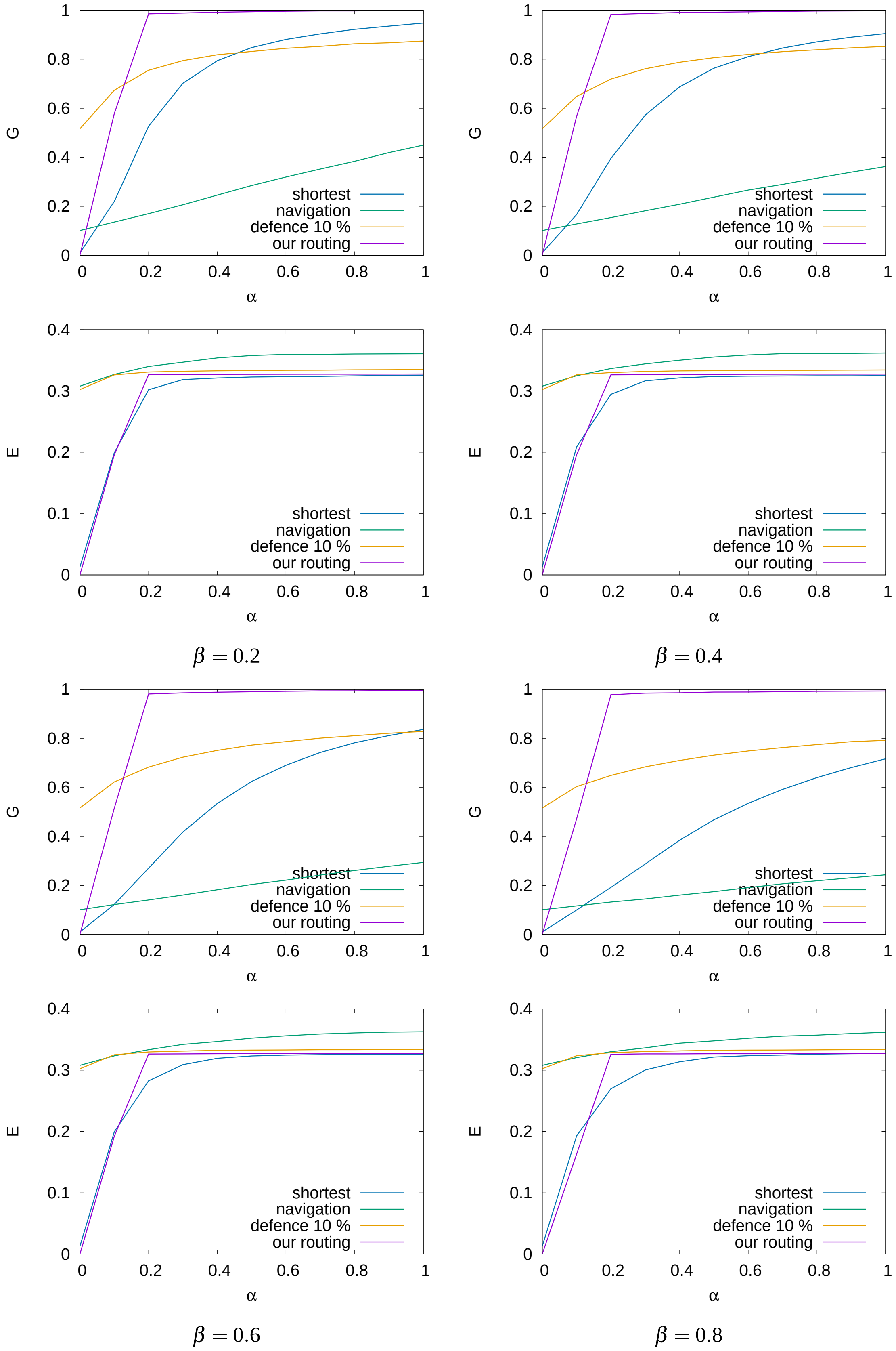

Figure S12. Comparison for the value of β in SF networks, Zhao's settig.

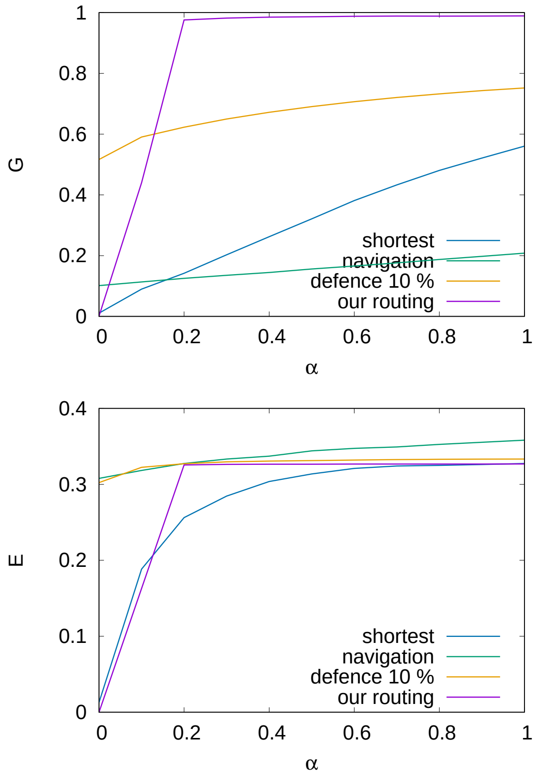

$\beta = 1.0$

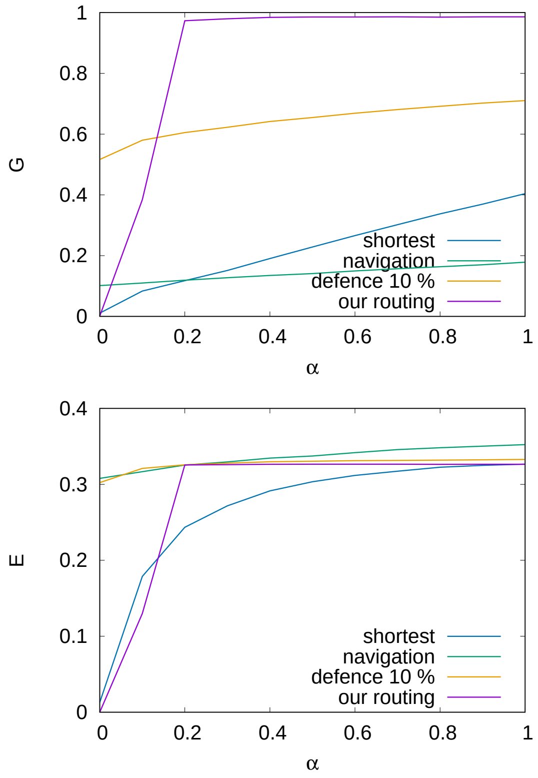

$\beta = 1.2$

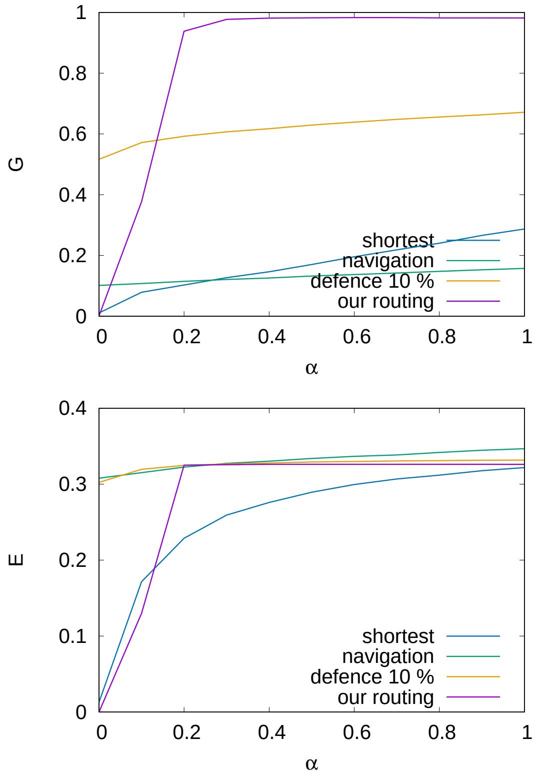

$\beta = 1.4$

Figure S13. (Continue) Comparison for the value of β in SF networks, Zhao's settig.

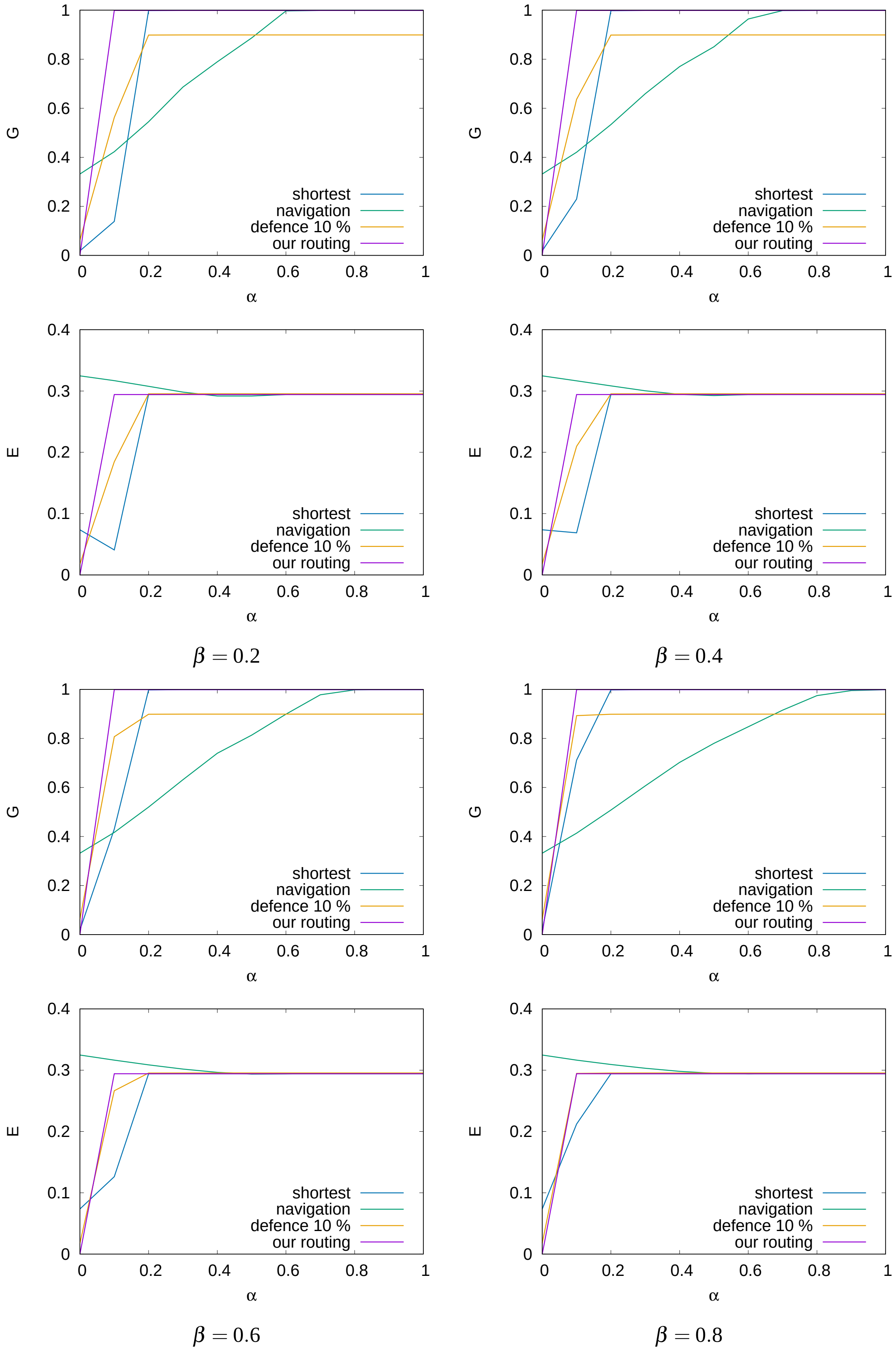

Figure S14. Comparison for the value of β in onion-like networks, Dou's settig.

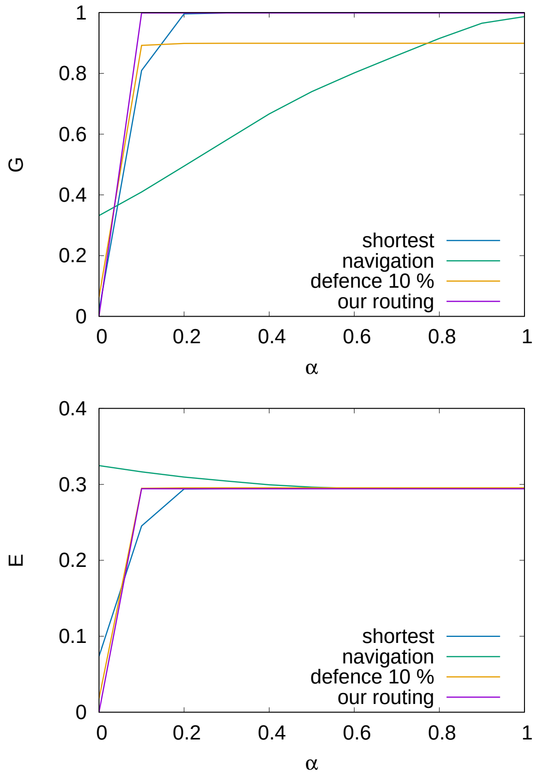

$\beta = 1.0$

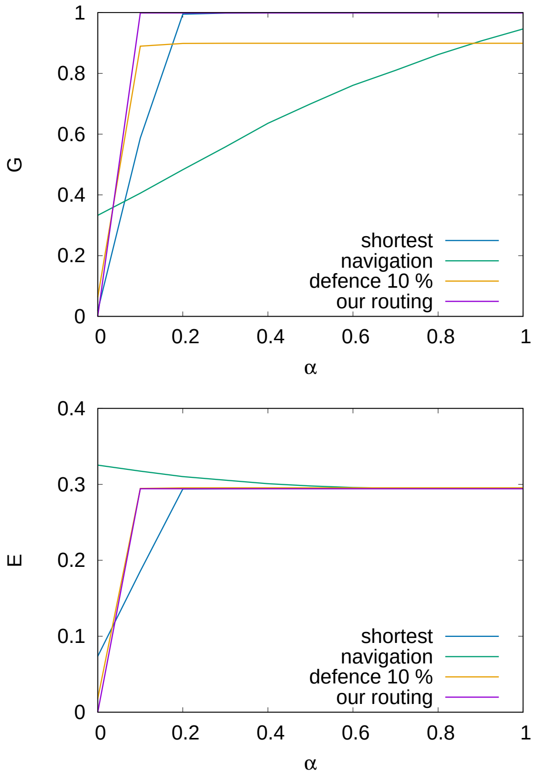

$\beta = 1.2$

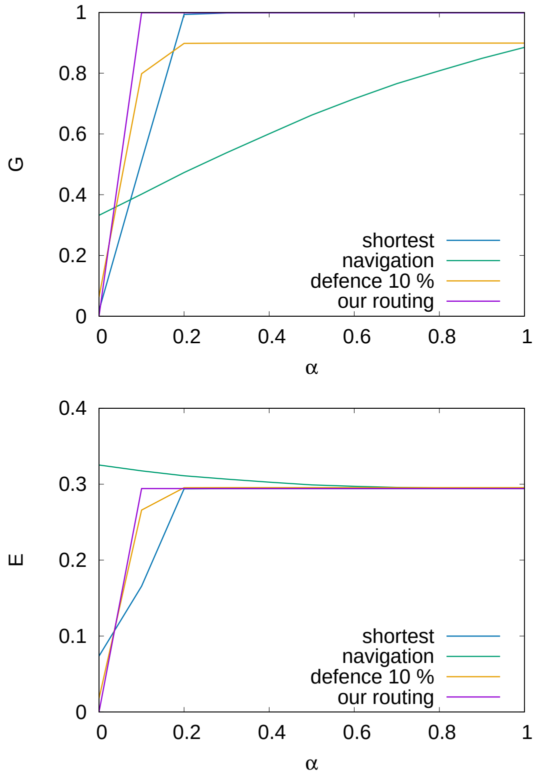

$\beta = 1.4$

Figure S15. (Continue) Comparison for the value of β in onion-like networks, Dou's settig.

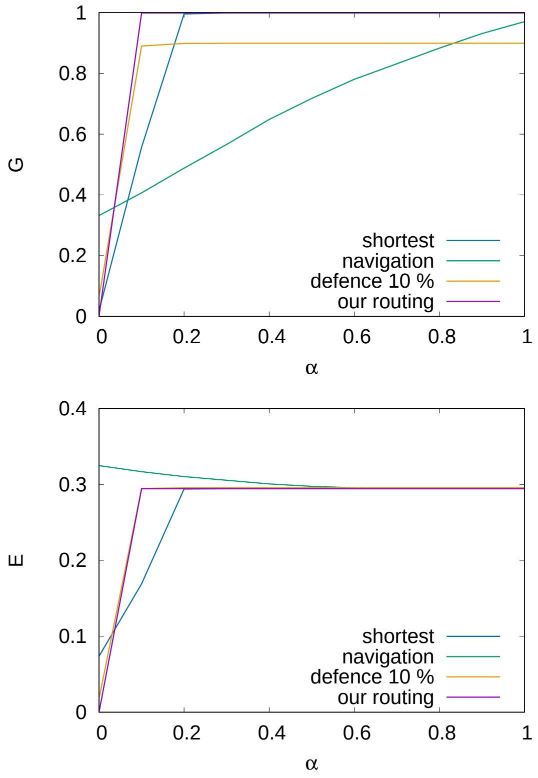

$\beta = 0.2$

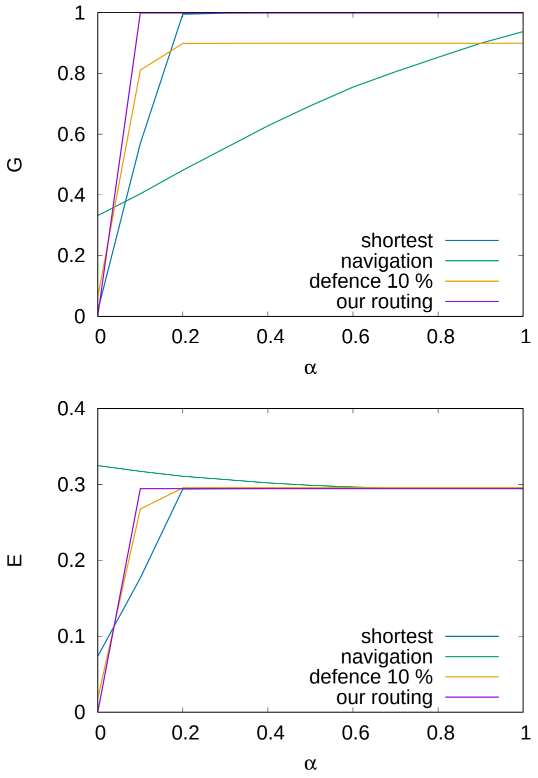

$\beta = 0.4$

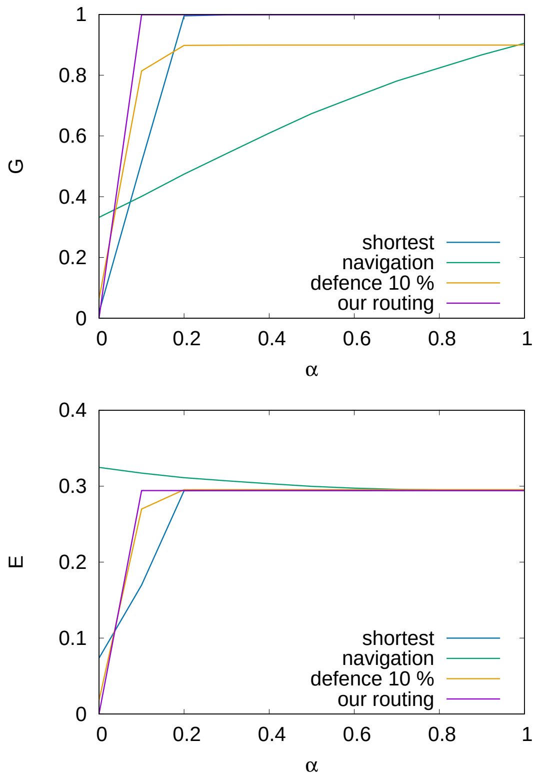

$\beta = 0.6$

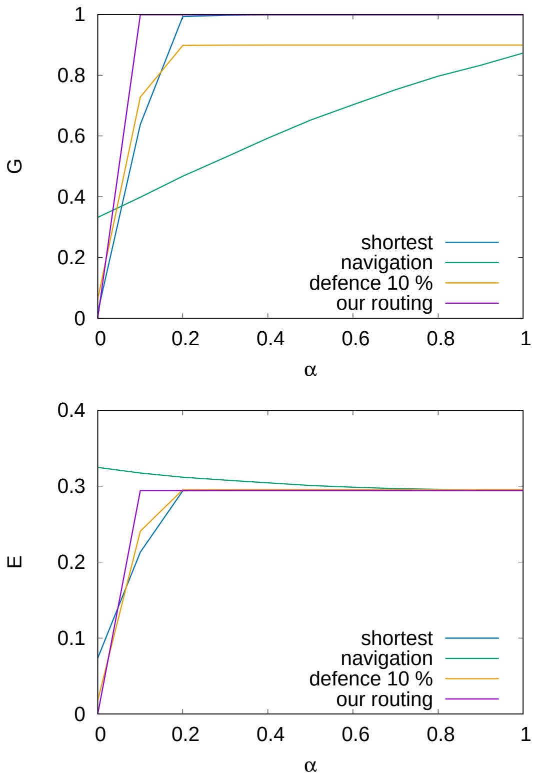

$\beta = 0.8$

Figure S16. Comparison for the value of β in onion-like networks, Zhao's settig.

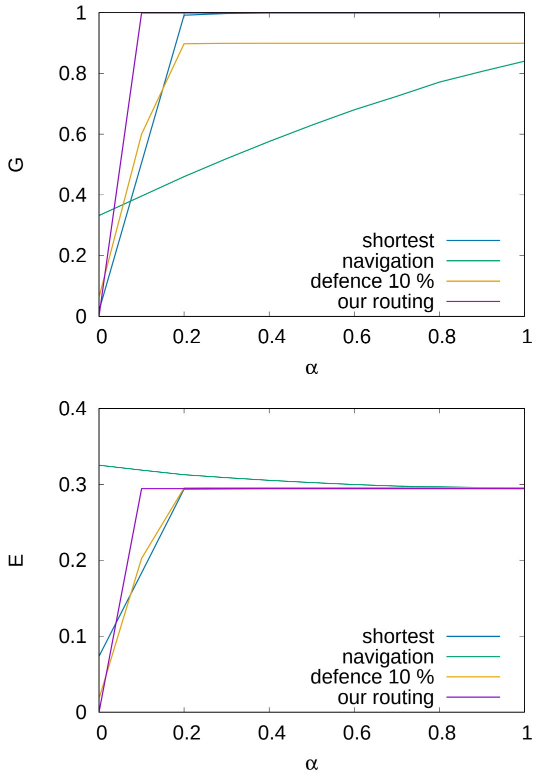

$\beta = 1.0$

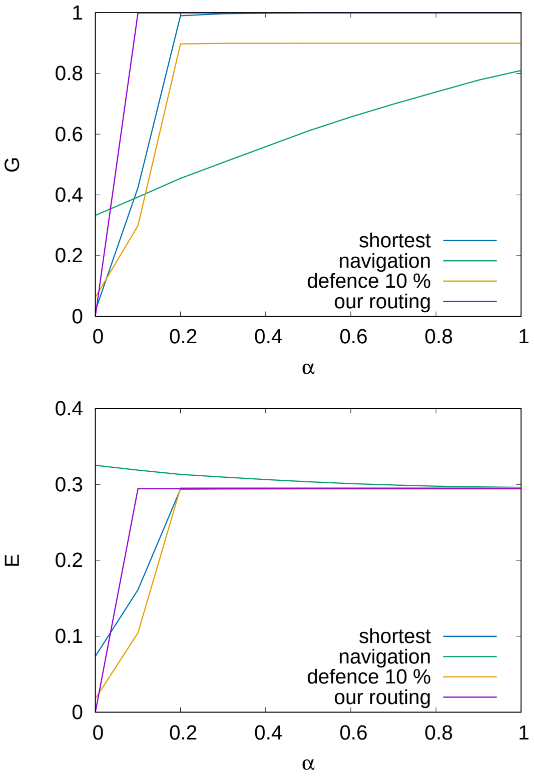

$\beta = 1.2$

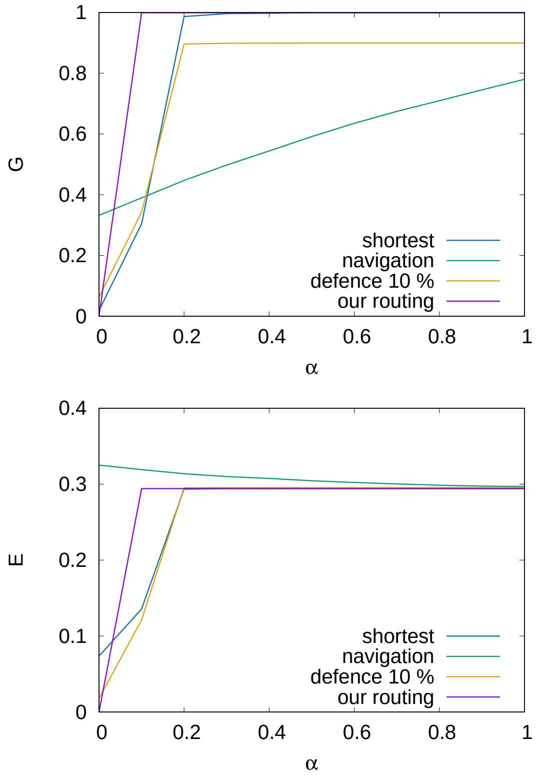

$\beta = 1.4$

Figure S17. (Continue) Comparison for the value of β in onion-like networks, Zhao's settig.

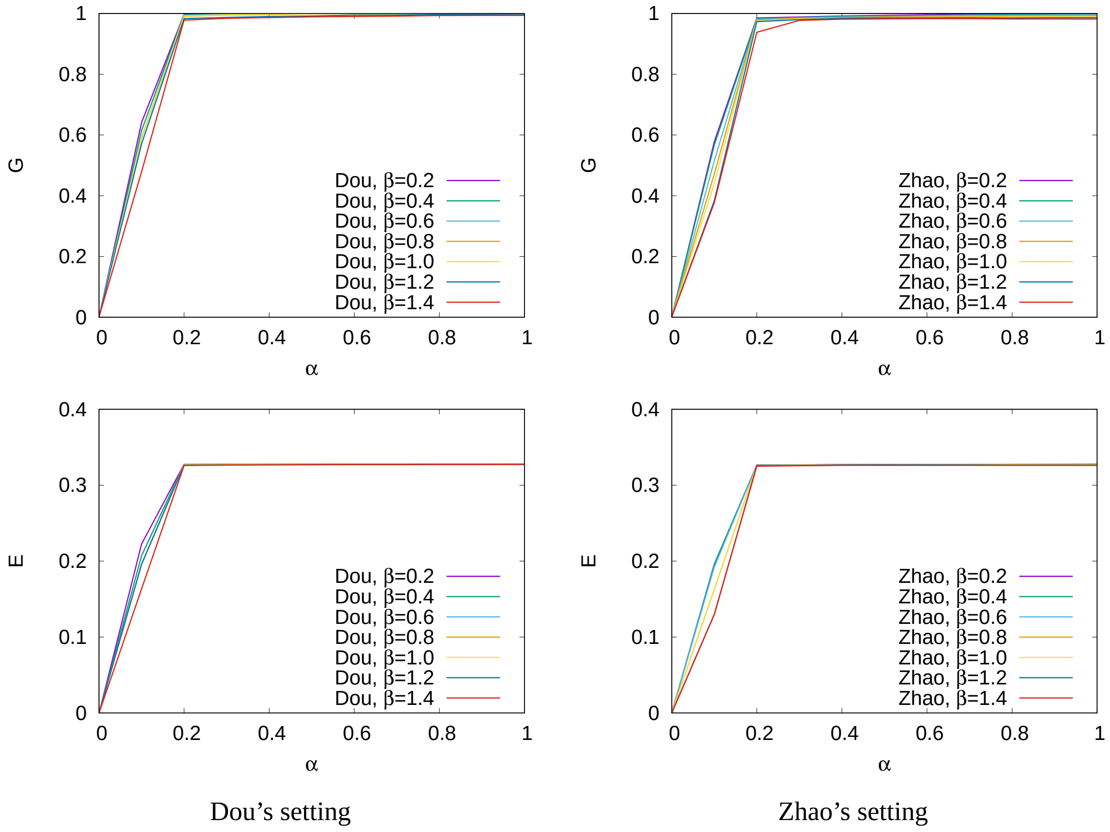

Figure S18. Comparison of the results by our routing for the value of β in SF networks.

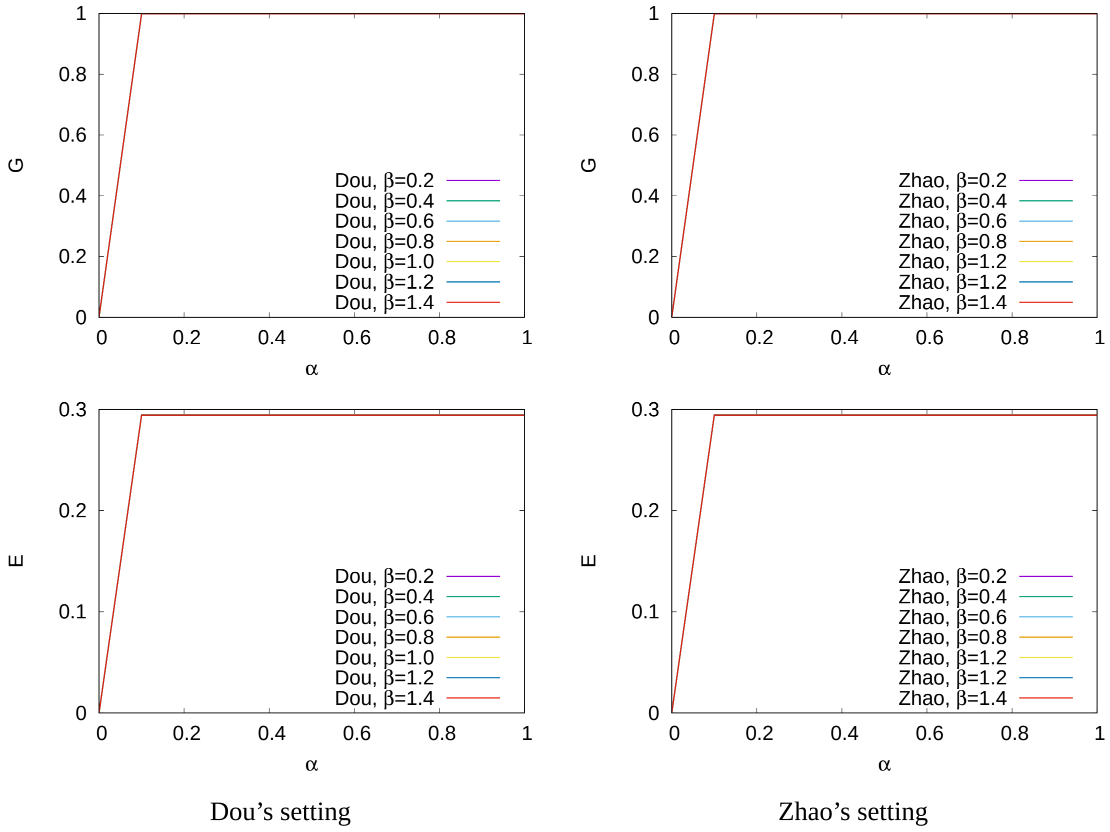

Figure S19. Comparison of the results by our routing for the value of β in onion-like networks.
